# Sources and Seasonal Variations of Per- and Polyfluoroalkyl Substances (PFAS) in Surface Snow in the Arctic

**DOI:** 10.1021/acs.est.4c08854

**Published:** 2024-11-26

**Authors:** William F. Hartz, Maria K. Björnsdotter, Leo W. Y. Yeung, Jack D. Humby, Sabine Eckhardt, Nikolaos Evangeliou, Ingrid Ericson Jogsten, Anna Kärrman, Roland Kallenborn

**Affiliations:** † Department of Earth Sciences, University of Oxford, South Parks Road, Oxford OX1 3AN, U.K.; ‡ Department of Arctic Geology, University Centre in Svalbard (UNIS), NO-9171 Longyearbyen, Svalbard, Norway; § NILU, Instituttveien 18, NO-2007 Kjeller, Norway; ∥ Man-Technology-Environment Research Centre (MTM), Örebro University, SE-701 82 Örebro, Sweden; ⊥ Institute of Environmental Assessment and Water Research (IDAEA-CSIC), C/Jordi Girona, 18-26, 08034 Barcelona, Catalonia, Spain; # Ice Dynamics and Paleoclimate, British Antarctic Survey, High Cross, Cambridge CB3 0ET, U.K.; ¶ Faculty of Chemistry, Biotechnology and Food Sciences (KBM), Norwegian University of Life Sciences (NMBU), NO-1432 Ås, Norway; ∇ University of the Arctic (UArctic), Yliopistonkatu 8, 96300 Rovaniemi, Finland

**Keywords:** atmospheric deposition, precursors, hydroxyl radicals, trifluoroacetic acid, solar flux, GenX, Svalbard

## Abstract

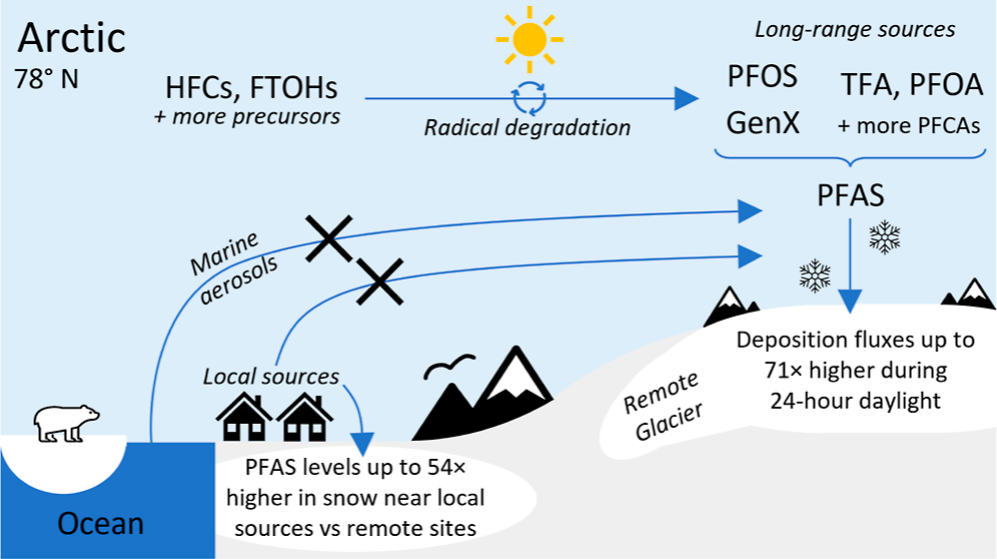

Per- and polyfluoroalkyl substances (PFAS) are persistent anthropogenic contaminants, some of which are toxic and bioaccumulative. Perfluoroalkyl carboxylic acids (PFCAs) and perfluoroalkyl sulfonic acids (PFSAs) can form during the atmospheric degradation of precursors such as fluorotelomer alcohols (FTOHs), *N*-alkylated perfluoroalkane sulfonamides (FASAs), and hydrofluorocarbons (HFCs). Since PFCAs and PFSAs will readily undergo wet deposition, snow and ice cores are useful for studying PFAS in the Arctic atmosphere. In this study, 36 PFAS were detected in surface snow around the Arctic island of Spitsbergen during January–August 2019 (i.e., 24 h darkness to 24 h daylight), indicating widespread and chemically diverse contamination, including at remote high elevation sites. Local sources meant some PFAS had concentrations in snow up to 54 times higher in Longyearbyen, compared to remote locations. At a remote high elevation ice cap, where PFAS input was from long-range atmospheric processes, the median deposition fluxes of C_2_–C_11_ PFCAs, PFOS and HFPO–DA (GenX) were 7.6–71 times higher during 24 h daylight. These PFAS all positively correlated with solar flux. Together this suggests seasonal light is important to enable photochemistry for their atmospheric formation and subsequent deposition in the Arctic. This study provides the first evidence for the possible atmospheric formation of PFOS and GenX from precursors.

## Abstract 2

PFAS deposition in the remote Arctic is highly seasonal, and widespread, as a result of atmospheric precursor degradations. Long-range atmospheric processes also deposit GenX in the Arctic.

## Introduction

Per- and polyfluoroalkyl substances (PFAS) are a diverse group of organofluorine contaminants, used in a variety of industrial and consumer applications.^1^ Their high mobility and environmental persistence, as well as their ability to undergo transformation in the environment, has led them to being detected ubiquitously in the environment.^2^ This includes, for example, Arctic biota and abiota.^3,4^ However, many PFAS are also known to be toxic and bioaccumulative.^5,6^

A large focus of research has been on perfluoroalkyl acids (PFAAs), including perfluoroalkyl carboxylic acids (PFCAs) and perfluoroalkyl sulfonic acids (PFSAs). Several long-range distribution mechanisms and their interactions explain the presence of PFAAs and other PFAS in the Arctic. These include ocean currents,^7,8^ marine aerosols,^9^ local sources,^3,10^ and the long-range atmospheric transport of volatile neutral PFAS (so-called precursors) followed by their atmospheric degradation and subsequent deposition.^11,12^

Measuring precipitation provides a route to understanding the atmospheric processes of PFAS.^13,14^ Several studies have used remote snow pits and ice cores to provide a record of the overall atmospheric processes over several years,^15−17^ whereas measuring just surface snow can provide information about the atmospheric conditions for the precipitation event sampled.^18^ Only one study has used surface snow to investigate seasonal variations in PFAA deposition. Björnsdotter et al. found that the deposition of C_2_–C_4_ PFCAs was highly seasonal in the Arctic.^19^ This study and others have linked trifluoroacetic acid (TFA) deposition with solar radiation.^20,21^ This is expected since solar radiation is able to initiate the atmospheric formation of radicals (e.g., hydroxyl radicals), which are known to degrade TFA precursors, including hydrofluorocarbons (HFCs),^22^ hydrochlorofluorocarbons (HCFCs),^23^ hydrofluoroethers (HFEs),^24^ and hydrofluoroolefins (HFOs).^25^ These were introduced in the 1990s as replacement products after the Montreal protocol banned ozone-depleting chlorofluorocarbons (CFCs).^26^ The atmospheric levels for many HFCs, and related compounds, are increasing,^27^ as is TFA deposition.^17,28,29^

Other precursors, such as fluorotelomer alcohols (FTOHs), *N*-alkylated perfluoroalkane sulfonamides (FASAs), perfluoroalkane sulfonamido ethanols (FASEs), and fluorotelomer acrylates (FTAs) are known to exist in the Arctic atmosphere,^30−34^ and they are also able to undergo radical-mediated degradation to PFAAs.^11,35−38^ The degradation of an *n*:2 FTOH will produce approximately equal molar quantities of the corresponding C_*n*_ and C_*n*+1_ PFCAs,^11^ and, using this approach (even:odd molar ratios), FTOHs have been found to be a major source of PFCA deposition in the remote Arctic.^12,16,17,39^ Nonetheless, local sources can also contribute to PFAS deposition in surface snow.^40^

In Svalbard (Norwegian Arctic), significant local sources in Longyearbyen and Ny-Ålesund, from landfill and firefighting training sites (FFTS) have been found.^3,10,40^ Ali et al. found high levels of PFAS runoff from the FFTS in Longyearbyen (Σ_14_PFAS = 57.4 ± 4.0 ng L^–1^), where aqueous film forming foams are used for firefighting training purposes. PFOS (35%) had the largest mass contribution in runoff followed by PFHxS (22%), PFHxA (18%), PFOA (11%), PFHpA (6%) and 6:2 FTSA (3%). In landfill leachate from Longyearbyen (Σ_14_PFAS = 643 ± 84 ng L^–1^), PFOS also had the highest mass contribution (48%) followed by C_6_–C_11_ PFCAs (43%) including PFOA (20%).

Nonetheless, Björnsdotter et al. found that the source of C_2_–C_4_ PFCAs in surface snow from Foxfonna, a high elevation ice cap outside of Longyearbyen (800 m.a.s.l., 16 km from Longyearbyen upwind of prevailing winds), was predominantly from long-range atmospheric processes, despite the location’s proximity to Longyearbyen settlement. The present study uses the same surface snow samples reported by Björnsdotter et al. (previously analyzed for C_1_–C_4_ PFAAs using supercritical fluid chromatography, SFC), to measure 38 further PFAS (using ultra performance liquid chromatography, UPLC) in surface snow in Svalbard (Norwegian Arctic). This study aims to investigate (i) the levels and seasonal variations of PFAS deposition in the Arctic (ii) the extent of PFAS contamination in surface snow from local sources in Longyearbyen and (iii) the atmospheric processes of PFAS in the remote Arctic.

## Materials and Methods

### Target Analytes

In total, 45 different PFAS were targeted as follows. PFAAs targeted were C_2_–C_14_, C_16_, and C_18_ PFCAs, and C_1_–C_10_ and C_12_ PFSAs. Fluorotelomers targeted were 4:2 FTSA, 6:2 FTSA, 8:2 FTSA, 6:2 FTUCA and 8:2 FTUCA. Further anionic target analytes included 6:2 Cl-PFESA, 8:2 Cl-PFESA, HFPO–DA (GenX), and ADONA. Nine neutral compounds were also targeted: FBSA, MeFBSA, FHxSA, MeFHxSA, FOSA, MeFOSA, EtFOSA, MeFOSE and EtFOSE. C_2_–C_4_ PFCAs and C_1_–C_4_ PFSAs were measured by SFC tandem mass spectrometry (MS/MS) and concentrations were previously reported by Björnsdotter et al.^19^ The other 38 anionic and neutral analytes were measured by UPLC MS/MS and are reported in the present study. A full list of names, abbreviations and instrument methods are described in Table S1.

### Sample Collection

**Figure 1:**
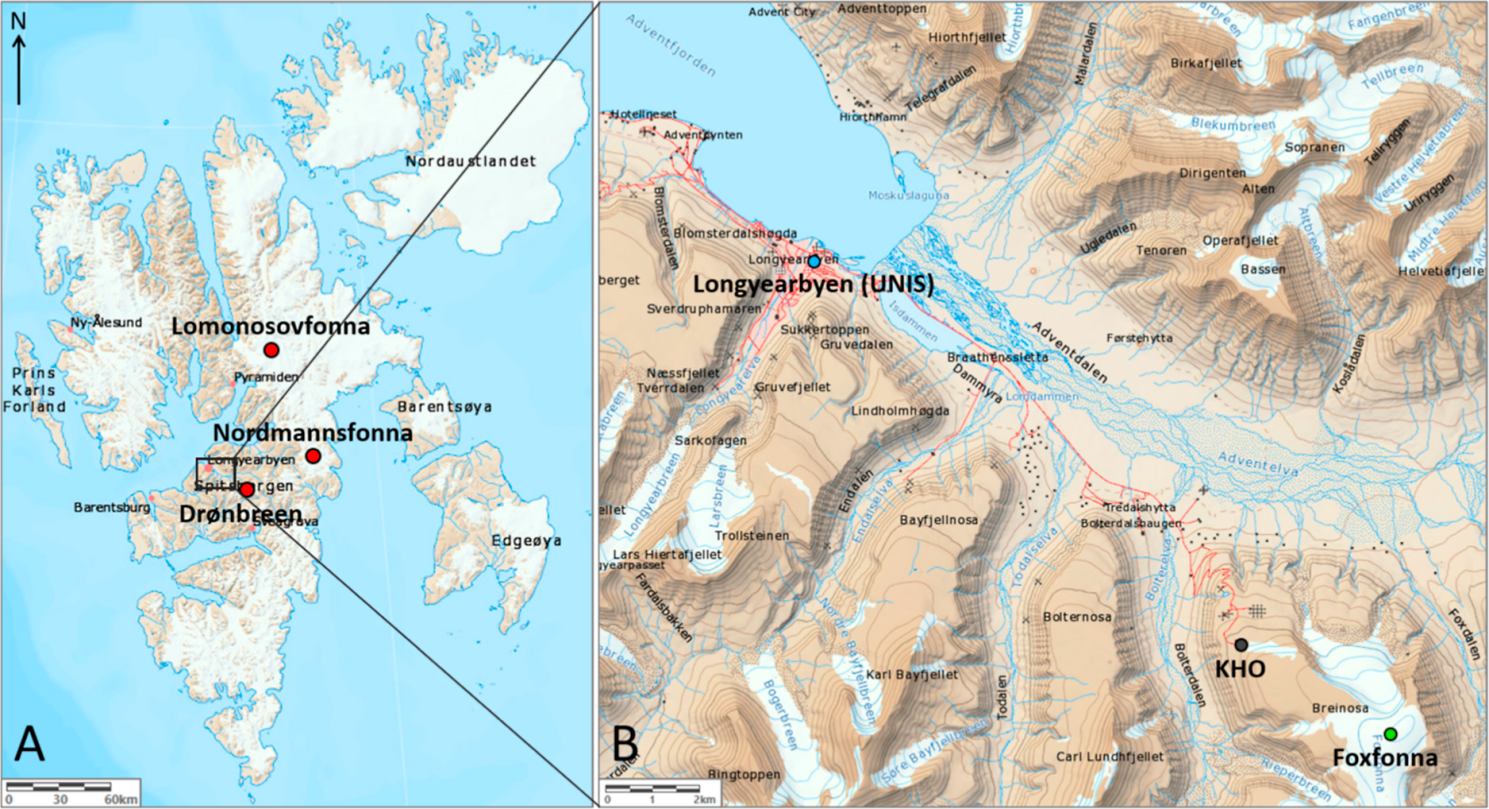
(A) The reference snow sampling locations on Spitsbergen glaciers: Lomonosovfonna (*n* = 2), Nordmannsfonna (*n* = 1), and Drønbreen (*n* = 1). (B) Sampling locations in Longyearbyen (UNIS, *n* = 8), near the KHO (*n* = 9) and on Foxfonna (*n* = 10). The map was reproduced from TopoSvalbard, Norwegian Polar Institute.

This study uses the same sample set previously used by Björnsdotter et al. which previously reported concentrations of C_1_–C_4_ PFAAs. A summary of the sampling procedure is described herein. In total six locations were chosen for surface snow sampling. Three of the sites offered a range of potential locally contaminated and background locations (Figure 1B) which were sampled several times during 2019 (*n* = 8–10). These three sites were in Longyearbyen (78°13.288′N 15°39.041′E, 13 m.a.s.l.), up the hill from the Kjell Henriksen Observatory (KHO; 78°08.807′N 16°02.781′E, 532 m.a.s.l.), and on the summit of Foxfonna ice cap (78°07.736′N 16°10.791′E, 800 m.a.s.l.).

The site on the summit of the Foxfonna ice cap was chosen as a potentially remote location owing to its high altitude (800 m.a.s.l.) and position eastwards upwind from the Longyearbyen settlement (16 km away). Close to Foxfonna (4.7 km away) sampling was also conducted 150 m uphill from the KHO. Sampling was also undertaken in the settlement of Longyearbyen (approximately 2400 inhabitants), outside the University Centre in Svalbard (UNIS). This was situated in approximately the center of the town, 360 m from the fjord, in a fenced off area such that the snow was not directly disturbed by pedestrians or traffic. Sampling was undertaken January–August 2019 at Foxfonna (samples Fox01–Fox10, *n* = 10), January–June 2019 at KHO (samples KHO01–KHO09, *n* = 9) and January–May 2019 at UNIS (samples UNIS01–UNIS08, *n* = 8). This represents the time of year when the seasonal light is changing from 24 h darkness in winter to 24 h daylight summer. 24 h daylight began on 19th April 2019 and continued for the rest of the sampling period.

Each snow sample represents a single precipitation event whereby post-depositional effects have been minimized. This was achieved by sampling as soon as safely possible after each chosen precipitation event. This was on average 1.5 days at the Foxfonna sampling site. Surface snow samples (*n* = 4) from three high elevation remote sites on glaciers were also collected (Figure 1A). These reference locations were selected as they would presumably receive PFAS input from long-range atmospheric processes only. Sampling was conducted during February–April 2019 on Drønbreen (*n* = 1, 78°06.185′N 16°39.182′E, 707 m.a.s.l.), Lomonosovfonna (*n* = 2, 78°49.454′N 17°26.253′E, 1198 m.a.s.l.), and Nordmannsfonna (*n* = 1, 78°15.894′N 18°23.717′E, 498 m.a.s.l.).

For each snow sample the upper 0–5 cm of the surface snow was collected into a precleaned high-density polyethylene barrel using a precleaned aluminum shovel. Sampling the upper 5 cm only was important to ensure consistency with respect to possible (i) photochemical transformations occurring because of light penetration into the snow surface, (ii) revolatilization of volatile PFAS back to the atmosphere after deposition and (iii) possible surface meltwater percolation mobilizing PFAS in the surface. All three factors are known to affect PFAS levels in snow.^34,41,42^ Hence, the 5 cm depth for sampling attempted to control for this with a consistent and reproducible sampling method. Due to windy conditions on Svalbard, it was assumed that the snowfall event was well mixed by snow blowing in the atmosphere and surface snow drifting before final deposition,^43^ and hence PFAS distribution in the freshly deposited snow layer after precipitation was homogeneous. Owing to weathering of the snow surface by wind between precipitation events, snow from the existing snow surface, established prior to the targeted precipitation event, was not significantly incorporated into the subsequently sampled snow layer following each chosen precipitation event. Therefore, the surface snow sampled overwhelmingly consisted of snow from the targeted precipitation events. The area sampled was 0.85–3.9 m^2^ (average 1.8 m^2^) for the 31 surface snow samples. After sampling, the barrel was sealed and transported back to UNIS, where the snow was then melted at 5 °C and bottled into precleaned polypropylene containers. Subsamples from the barrel were also taken for major ion analysis.

### Sample Extraction and PFAS Analysis

All samples were extracted according to the methods described in detail by Björnsdotter et al.^19^ In summary, the snow samples (approximately 2200 mL of melted snow) were first filtered, and filters extracted in MeOH, and then the extracts combined with the filtered snow sample prior to PFAS extraction. The samples were then extracted by weak anion exchange solid-phase extraction (Oasis WAX, Waters Corporation, Milford, USA) following the ISO25101 method with some modifications.^44^ After extraction, two fractions containing target analytes were eluted from the cartridge. The first fraction containing neutral target analytes was eluted by adding 4 mL methanol. The second fraction containing anionic target analytes was eluted by adding 4 mL 0.1% ammonium hydroxide in methanol. The anionic fraction has already been analyzed by SFC-MS/MS for quantification of C_1_–C_4_ PFAAs. Their concentrations and instrument methods are reported by Björnsdotter et al.^19^ Quantification of the remaining 38 anionic and neutral PFAS by UPLC-MS/MS, as well as full details of the chemicals and reagents, quality control and quality assurance and MQLs (method quantification limits) is reported in the Supporting Information (pages S1–S4). Perfluoromethyl branched isomers of PFHxS and PFOS were quantified according to the methods described by Hartz et al.^17^

### Data Treatment

Concentrations of PFAS are reported in pg L^–1^ (Tables S7–S10). To remove the effects of variations in the magnitude of each precipitation event, PFAS concentrations (in pg L^–1^) were multiplied by the total precipitation for each snowfall event (in kg m^–2^) to yield PFAS deposition fluxes (in pg m^–2^ per precipitation event). The total precipitation for each precipitation event (see Table S17) was calculated by summing the precipitation per hour, throughout the timespan of each precipitation event at each sampling site, according to the ERA5 hourly reanalysis model from the European Centre for Medium-Range Weather Forecasts (ECMWF).^45^ Using precipitation to calculate deposition fluxes, rather than in situ snow accumulation, meant that the total PFAS delivered to the Arctic environment per precipitation event could be calculated, rather than localized information about PFAS accumulation on the glacier/sampling site. PFAS deposition fluxes are reported for all compounds in this manuscript, and also for C_1_–C_4_ PFAAs (previously reported by Björnsdotter et al.),^19^ whose deposition fluxes we have improved upon based on this new approach (Tables S11–S14). Results are also compared to Na^+^ ion concentrations, measured using ion chromatography, and solar fluxes (Table S17), calculated using the Lagrangian Particle Dispersion Model FLEXPART version 10.4,^46^ the methods and calculations for which are described in the Supporting Information (pages S4–S6). Spearman rank correlation coefficients (*r*) were used to investigate correlations. Tests for statistical significance were performed using a two-tailed Student’s *t* test (p). For calculations in Table 2, concentrations < MQL were substituted with MQL/2.

## Results and Discussion

### Concentrations and Fluxes of PFAS in Surface Snow

Table 1 outlines the detection of the 45 PFAS targeted by this study and Björnsdotter et al.^19^ C_5_–C_8_ PFCAs, PFHxS, PFOS, 6:2 FTSA and FHxSA were detected in all surface snow samples, in addition to TFA, PFPrA and TFMS. The widespread detection (75–100%), including at the reference locations, of C_2_–C_9_ PFCAs, PFUnDA, PFTrDA, HFPO–DA (GenX), TFMS, PFHxS, PFOS, 6:2 FTSA, FBSA, FHxSA and FOSA, suggests that long-range atmospheric processes enable their deposition in the remote Arctic. The PFAS observed broadly agree with two remote Arctic ice cores, from Lomonosovfonna, Svalbard (Table 1) and the Devon Ice Cap, Canada, which found widespread detection of C_2_–C_11_ PFCAs, PFOS and FOSA.^16,17,29^ Further information on the concentrations and deposition fluxes of the 45 targeted PFAS can be found in Tables S7–S14. A full list of names, abbreviations and instrument methods are described in Table S1.

**Table 1:** Detection Frequency (%) of PFAS at the Threes Snow Sampling Sites, the Reference Sites and in a Svalbard Ice Core from Lomonosovfonna (Lomo, Reported by Hartz et al. 2023^17^)^a^

	PFAS	UNIS, Longyearbyen	KHO	Foxfonna	Reference sites	Svalbard Ice Core (Lomo)
Perfluoroalkyl Carboxylic Acids (PFCAs)	TFA	**100**	**100**	**100**	**100**	**100**
	PFPrA	**100**	**100**	**100**	**100**	**100**
	PFBA	**100**	**100**	**90**	**100**	**100**
	PFPeA	**100**	**100**	**100**	**100**	**100**
	PFHxA	**100**	**100**	**100**	**100**	**100**
	PFHpA	**100**	**100**	**100**	**100**	**100**
	PFOA	**100**	**100**	**100**	**100**	**100**
	PFNA	**100**	**100**	**90**	**100**	**100**
	PFDA	**100**	44	70	25	**100**
	PFUnDA	**100**	**78**	70	**100**	**100**
	PFDoDA	**75**	**78**	50	**75**	**82**
	PFTrDA	**100**	**89**	**90**	**75**	**88**
	PFTDA	**100**	44	40	**75**	18
	PFHxDA	**88**	0	0	0	12
	PFOcDA	50	0	0	0	0
						
Perfluoroalkyl Sulfonic Acids (PFSAs)	TFMS	**100**	**100**	**100**	**100**	n/a
	PFEtS	**100**	22	0	0	0
	PFPrS	0	0	0	0	0
	PFBS	63	0	10	0	24
	PFPeS	13	11	0	0	6
	PFHxS	**100**	**100**	**100**	**100**	65
	PFHpS	38	0	0	0	0
	PFOS	**100**	**100**	**100**	**100**	**82**
	PFNS	0	0	0	0	0
	PFDS	0	0	0	0	0
	PFDoDS	0	0	0	0	0
						
Neutral PFAS	FBSA	**100**	**89**	**80**	**75**	**82**
	MeFBSA	**100**	22	50	**100**	29
	FHxSA	**100**	**100**	**100**	**100**	6
	MeFHxSA	0	0	0	0	0
	FOSA	**100**	**89**	**90**	**75**	59
	MeFOSA	0	0	0	0	0
	EtFOSA	0	11	10	0	6
	MeFOSE	38	33	40	**75**	24
	EtFOSE	63	44	40	0	35
						
Other PFAS	4:2 FTSA	0	0	0	0	0
	6:2 FTSA	**100**	**100**	**100**	**100**	41
	8:2 FTSA	25	0	10	0	0
	6:2 FTUCA	38	**78**	**80**	**100**	35
	8:2 FTUCA	25	22	0	50	6
	PFECHS	**75**	11	0	0	0
	6:2 Cl-PFESA	0	0	0	0	0
	8:2 Cl-PFESA	0	0	0	0	0
	ADONA	0	11	10	0	0
	HFPO-DA (GenX)	**75**	**78**	**90**	**75**	0

a Results for C_1_–C_4_ PFAAs have previously been reported by Björnsdotter et al.^19^ Detection frequencies ≥75% are in bold.

The widespread detection of C_2_–C_11_ PFCAs in surface snow likely comes from the radical-mediated atmospheric degradation of precursor compounds.^16,17,29^ The widespread detection of HFPO-DA and 6:2 FTSA in surface snow, including at the reference sites, suggests that long-range atmospheric processes can also contribute to their deposition in the Arctic. 6:2 fluorotelomer sulfonamide alkylamine and 6:2 fluorotelomer sulfonamide were previously suggested as possible atmospheric precursors to 6:2 FTSA.^17^

At the reference sites, there was frequent detection of FBSA (75%), FHxSA (100%) and FOSA (75%), MeFBSA (100%) and MeFOSE (75%). The detection of FASAs and FASEs on these high elevation remote glaciers is in agreement with the detection of these compounds in snow on glaciers near Ny-Ålesund,^34^ and an ice core from Lomonosovfonna, Svalbard (Table 1), and could be explained by their direct long-range transport from source regions. This confirms that these known PFAA precursors exist in the Arctic atmosphere.^36,37^

6:2 FTUCA was previously identified as an atmospheric degradation product of 6:2 FTOH in a Svalbard ice core.^17^ 6:2 Fluorotelomer carboxylic acid (6:2 FTCA) will eliminate HF to form 6:2 FTUCA in the environment (e.g., during post-deposition processes) or during extraction/analysis (e.g., during the preparation of the extracts in MeOH).^47,48^ Hence, the detection of 6:2 FTUCA is evidence for the atmospheric oxidation of the terminal alcohol of 6:2 FTOH to 6:2 FTCA. This study detected 6:2 FTUCA with the highest frequency at the reference sites (100%, elevations 498–1198 m.a.s.l.), followed by Foxfonna (80%, 800 m.a.s.l.), KHO, (78%, 532 m.a.s.l.), and then at UNIS (38%, 13 m.a.s.l.). The lesser detection frequency at lower elevation sites could be due to higher rates of post-deposition degradation at lower elevations. 6:2 FTCA has a –CH_2_– moiety that will be vulnerable to further degradation, for example, as part of post-depositional processes. This could also explain its low detection frequency in the Lomonosovfonna ice core (35%), where there are significant post-deposition processes such as firnification, meltwater percolation and seasonal light penetration into the upper surface of the glacier.

Like TFMS,^19^ PFHxS and PFOS were also detected in all snow samples. This study also quantified perfluoromethyl branched isomers of PFHxS and PFOS (Tables S15 and S16). Like the Lomonosovfonna ice core, snow in this study was dominated by linear-PFHxS samples and it was the only isomer detected in 97% of all surface snow samples. PFHxS produced by electrochemical fluorination (ECF) is known to produce 18% branched PFHxS isomers.^49^ Therefore, low levels of linear-PFHxS in the surface snow likely mean that any branched isomers were < MQL. In contrast, branched isomers of PFOS were observed in every sample as well as linear-PFOS. The percentage of linear isomers of PFOS at Foxfonna, KHO, UNIS and the reference sites was 82 ± 4%, 79 ± 3%, 83 ± 3%, and 81 ± 1% respectively. These results match the Lomonosovfonna ice core (81 ± 6%),^17^ but were slightly higher than runoff from the FFTS (69 ± 1%), landfill leachate (73 ± 1%), and the river in Longyearbyen (74 ± 1%).^3^ Regardless, all these locations confirm ECF manufacture as a major contributing source.^49,50^

Herein, spatial, and seasonal trends are investigated for those compounds with acceptable extraction efficiencies. These were for C_2_–C_11_ PFCAs, FBSA, TFMS, PFHxS, PFOS and HFPO-DA (GenX), whose extraction efficiencies averaged 60 ± 21% (see Tables S3–S5).

### Comparison between Snow Sampling Locations and the Lomonosovfonna 2019 Ice Core

**Figure 2:**
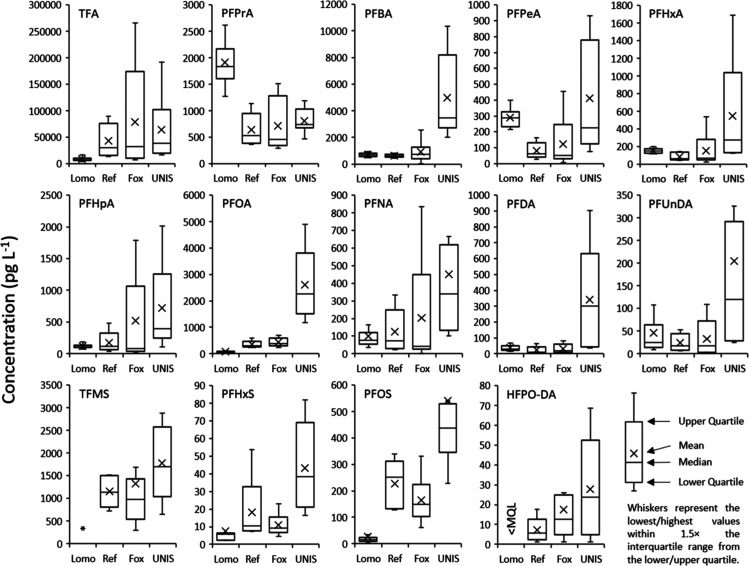
Boxplot to show the concentrations (pg L^–1^) of C_2_–C_11_ PFCAs, TFMS, PFHxS, PFOS and HFPO-DA (GenX) in an ice core from Lomonosovfonna (Lomo) spanning 2006–2019 (Hartz et al.^17^) and surface snow (this study) from reference sites (Ref), Foxfonna (Fox) and Longyearbyen (UNIS). Concentrations of TFMS and C_2_–C_4_ PFCAs in the surface snow have previously been reported by Björnsdotter et al.^19^ *Not able to be reported in the Lomonosovfonna ice core.

An ice core from Lomonosovfonna, Svalbard (spanning 2006–2019) has been analyzed for the same 45 PFAS as in this study.^17^ The concentrations of C_2_–C_11_ PFCAs, TFMS, PFHxS, PFOS and HFPO-DA in the Lomonosovfonna ice core were compared with the concentrations of these same PFAS in surface snow from the reference sites (Ref), Foxfonna and Longyearbyen settlement (UNIS) reported in this study and by Björnsdotter et al. (Figure 2). The Lomonosovfonna ice core has likely only received PFAS input from long-range atmospheric sources.^17^ By comparing the levels of different PFAS at these four locations, this can be used to understand whether each site receives PFAS input predominantly from local sources, or from long-range atmospheric processes alone. Björnsdotter et al. found that surface snow at UNIS (Longyearbyen) was influenced by local sources of PFBA, PFEtS and PFBS, and that Foxfonna represented a suitable location for understanding the long-range atmospheric processes of TFA, PFPrA, PFBA, and TFMS.

The median concentrations of C_5_–C_11_ PFCAs, PFHxS and PFOS were 2.3–22 times higher, when comparing snow at UNIS with the reference sites (Figure 2 and Table 2). This indicates that surface snow in Longyearbyen has been influenced by local sources. This reflects existing knowledge about PFAS sources in Longyearbyen.^3,10,40^ In contrast, median concentrations of C_5_–C_11_ PFCAs were similar on Foxfonna when compared to the reference sites (0.77–1.4 times higher, Table 2). Concentrations of PFHxS and PFOS were 1.0 and 0.77 times higher on Foxfonna compared to the reference sites. This suggests that Foxfonna receives input of these PFAS predominantly from long-range atmospheric processes, as was found previously for TFMS and C_2_–C_4_ PFCAs.^19^ This would be expected since the sampling site (800 m.a.s.l.) is located above the atmospheric boundary layer and is 16 km upwind from Longyearbyen with respect to the prevailing wind direction. To support this observation further, TFA (C_2_) made up 95% and 96% of the mass of C_2_–C_11_ PFCAs at the reference sites and on Foxfonna respectively, whereas other PFCAs individually contributed <1.7%. In contrast, TFA made up 85% of C_2_–C_11_ PFCAs in surface snow in Longyearbyen, with other PFCAs individually contributing up to 6.7%. These differences and similarities in PFCA distribution suggest that similar atmospheric processes are contributing to PFCA deposition at the reference sites and Foxfonna, whereas different sources exist for the surface snow in Longyearbyen.

**Table 2:** Results from Investigations into PFAS Atmospheric Processes for Surface Snow^a^

	Times higher at UNIS vs Ref^b^	Times higher at Foxfonna vs Ref^c^	Times higher median fluxes during 24 h daylight^d^	Correlation with Solar Flux^e^	Correlation with Na^+^^f^	Percentage of the total flux in samples Fox09 and Fox10 (%)^g^	Even:odd molar ratios between 0.5 and 2 (%)^h^
TFA	1.6	1.3	71	***r* = 0.88,** ***p* < 0.01**	*r* = 0.33, *p* = 0.35	62	0
PFPrA	1.6	1.0	28	***r* = 0.83,*p* < 0.01**	*r* = 0.55, *p* = 0.10	53	
PFBA	6.0	1.3	7.6	***r* = 0.87,*p* < 0.01**	*r* = 0.25, *p* = 0.49	41	0
PFPeA	3.8	0.88	29	***r* = 0.93,*p* < 0.01**	*r* = 0.21, *p* = 0.56	55	
PFHxA	4.8	1.2	31	***r* = 0.90,*p* < 0.01**	*r* = 0.18, *p* = 0.63	50	50
PFHpA	3.8	0.79	68	***r* = 0.83,*p* < 0.01**	*r* = 0.32, *p* = 0.37	75	
PFOA	8.0	1.3	13	***r* = 0.75,*p* < 0.05**	*r* = 0.54, *p* = 0.11	52	33
PFNA	6.3	0.77	39	***r* = 0.85,*p* < 0.01**	*r* = 0.33, *p* = 0.35	65	
PFDA	22	1.3	23	***r* = 0.93,*p* < 0.01**	*r* = 0.30, *p* = 0.40	53	86
PFUnDA	9.4	1.4	61	***r* = 0.93,*p* < 0.01**	*r* = 0.15, *p* = 0.68	55	
TFMS	1.7	1.0	8.8	*r* = 0.45, *p* = 0.19	*r* = 0.35, *p* = 0.33	61	n/a
PFHxS	4.2	1.0	7.3	*r* = 0.58, *p* = 0.08	*r* = 0.41, *p* = 0.24	45	n/a
PFOS	2.3	0.77	22	***r* = 0.73,*p* < 0.05**	*r* = 0.31, *p* = 0.38	49	n/a
FBSA	54	7.0	190	***r* = 0.82,*p* < 0.01**	*r* = −0.57, *p* = 0.08	29	n/a
HFPO-DA (GenX)	5.3	2.8	43	***r* = 0.89,*p* < 0.01**	*r* = 0.04, *p* = 0.91	32	n/a

a Results for TFMS and C_2_–C_4_ PFAAs were previously reported by Björnsdotter et al.^19^ Statistically significant spearman correlations (*r*) are in bold (*p* < 0.05).

b The median times higher concentrations at UNIS compared to the reference sites (Ref).

c The median times higher concentrations at Foxfonna compared to the reference sites (Ref).

d The median times higher deposition fluxes of PFAS during months with 24 h daylight at Foxfonna (Fox06–Fox10 compared to Fox01–Fox05).

e Correlations of PFAS deposition fluxes with solar flux for Foxfonna.

f Correlations of PFAS concentrations with Na^+^ (a marine aerosol proxy) for Foxfonna.

g The percentage of PFAS that was deposited in two samples during June and August (Fox09 and Fox10) across all 10 samples from Foxfonna.

h The percentage of PFCA even:odd molar ratios that were between 0.5 and 2, indicating a possible FTOH source to Foxfonna.

Variations in the range of concentrations between different snow sampling sites for several PFCAs, PFHxS and PFOS could be due to slight variations in the time of year in which sampling was conducted (Ref = February–April, Fox = January–August, UNIS = January–May). Large seasonal variations in C_2_–C_4_ PFCA deposition in the Arctic is known.^19^ Lower TFA concentrations (and other PFAS such as PFOA and PFOS) in the Lomonosovfonna ice core could be due to losses to glacier runoff or percolation to deeper layers in the firn. Indeed, TFA and PFOS are known to be mobile during meltwater percolation.^17^ Alternatively, variations between the PFAS concentrations in the ice core and the surface snow (e.g., lower TFA and higher PFPrA concentrations) could also be in part due to variations in the historical atmospheric levels of precursors during 2006–2019 (i.e., the timespan of the ice core),^17,27,51^ in contrast to atmospheric levels in 2019 alone, when the surface snow sampling in this study was conducted.

HFPO-DA (GenX) was detected at most surface snow sampling locations (75–90%) but was not detected in the Lomonosovfonna ice core.^17^ It was however detected in surface snow at the same location on Lomonosovfonna (3.3 and 5.7 pg L^–1^). This could indicate that post-depositional processes degrade HFPO-DA, or effective dilution from low HFPO-DA concentration snow during midwinter brings concentrations in the ice core below the MQL (2.3 pg L^–1^). HFPO-DA concentrations in snow from the reference sites and Foxfonna (<2.3–60.5 pg L^–1^) were similar to surface seawater from the Arctic Ocean (<6.0–70 pg L^–1^, median = 18.5 pg L^–1^).^52^ This suggests that these sites represented a long-range signal for HFPO-DA deposition. Based on concentrations, contamination of surface snow in Longyearbyen from local sources of HFPO-DA was less significant than local sources of PFOA or PFOS (Figure 2).

It was unclear why PFOA median concentrations were lower in the ice core (74.0 pg L^–1^), but higher in the reference sites (283 pg L^–1^) and on Foxfonna (379 pg L^–1^). Furthermore, two surface snow samples collected during April 2019 from Lomonosovfonna had PFOA concentrations of 305 and 243 pg L^–1^, which were also higher than the concentrations in the ice core drilled at the same site (39.8–112 pg L^–1^).^17^ 82% of the PFOA:PFNA ratios were between 0.5 and 2 indicating a FTOH precursor source for the ice core. However, for the reference sites (including surface snow from Lomonosovfonna) and on Foxfonna, just 20% and 33% of the ratios fell between 0.5 and 2, respectively (for those samples > MQL). The specific reasons for the differences in these concentrations and PFOA:PFNA ratios are unclear, but it points toward a possible division in the processes that exist for ice core and surface snow samples for PFOA (see Section Seasonal Variations in PFAS deposition in the Arctic). Indeed, an ice core provides a record of the overall atmospheric process over several years, whereas surface snow samples provide only information about the atmospheric conditions associated with the individual precipitation event sampled.

### Seasonal Variations in PFAS Deposition in the Arctic

**Figure 3:**
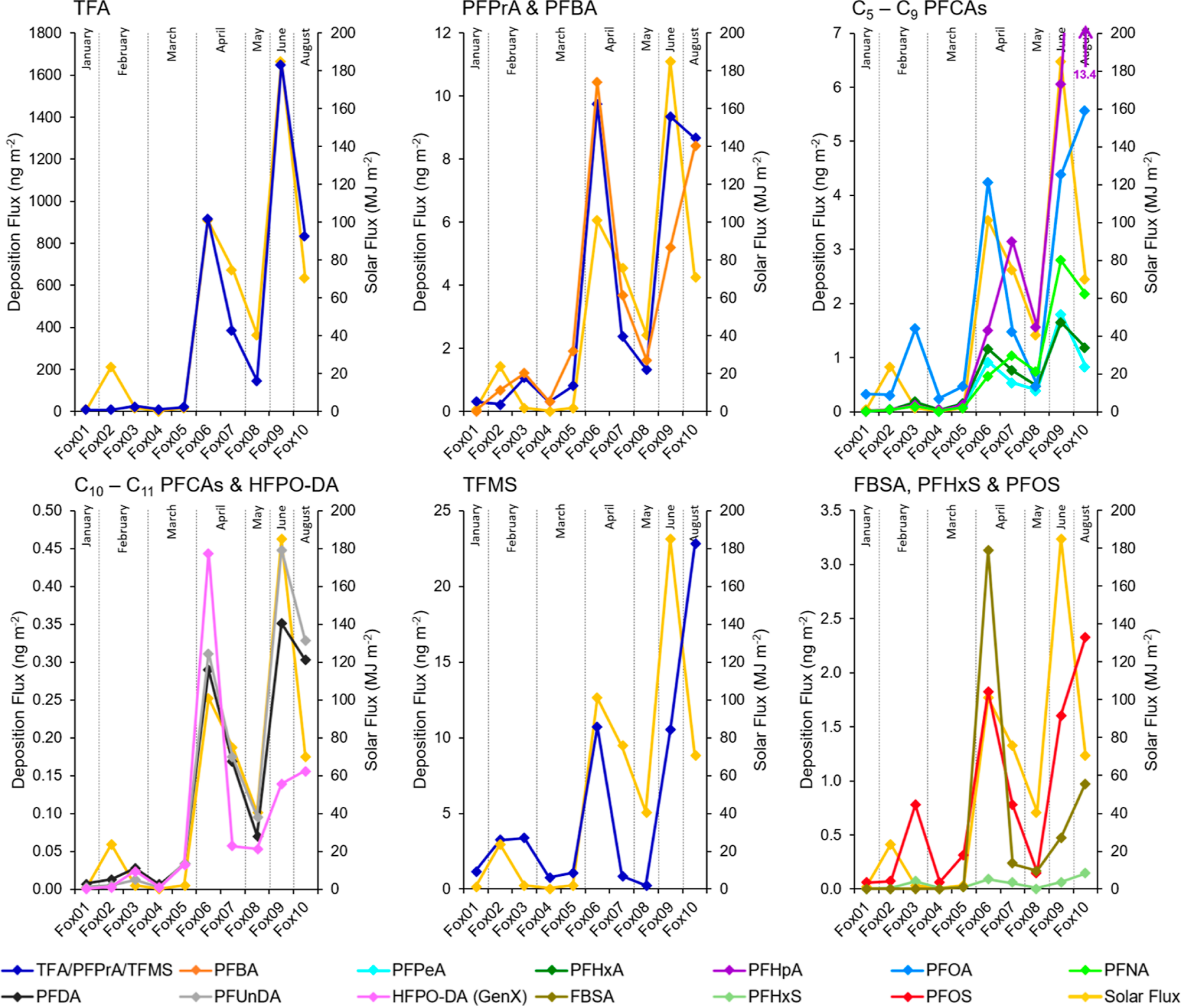
Deposition fluxes per precipitation event (ng m^–2^) of TFMS, PFHxS, PFOS, FBSA, HFPO-DA (GenX) and C_2_–C_11_ PFCAs in surface snow on Foxfonna during January to August 2019, plotted alongside the solar flux (MJ m^–2^).

As discussed previously, Foxfonna represented a location receiving input predominantly from long-range atmospheric processes of TFMS, PFHxS, PFOS, C_2_–C_7_ and C_9_–C_11_ PFCAs, HFPO-DA and possibly also PFOA. Foxfonna also had the longest sampling time series (January–August 2019) of the sites in this study. This offered the best opportunity to understand the seasonal variations in PFAS deposition in the remote Arctic (Figure 3). Seasonal variations in the fluxes of PFAS in surface snow in Longyearbyen are discussed further in the Supporting Information (pages S7–S8).

Together with the results from Björnsdotter et al.,^19^ C_2_–C_11_ PFCAs were found to have statistically significantly correlations with solar flux (0.75 ≤ *r* ≤ 0.93, *p* < 0.05) and their median deposition fluxes were 7.9–71 times higher during 24 h daylight (April–August) compared to snow samples collected during days with complete or partial darkness (Fox06–Fox10 compared to Fox01–Fox05, Table 2). This was also found to be the case for FBSA (*r* = 0.82, *p* < 0.01), PFOS (*r* = 0.73, *p* < 0.05) and HFPO-DA (*r* = 0.89, *p* < 0.01), whose median fluxes were 190, 22, and 43 times higher respectively during 24 h daylight. This suggests that solar radiation was important for the initiation of radical based chemistry (e.g., hydroxyl radicals) for the atmospheric formation of PFCAs, PFOS and HFPO-DA from precursors in the Arctic. Indeed, those samples collected during January–March (Fox01–Fox05) were from precipitation events when Svalbard and the High Arctic was largely isolated from downward UV radiation (Figure S3b), required for the atmospheric formation of hydroxyl radicals. The fluxes of C_6_–C_11_ PFCAs and several other PFAS were found to be highest in samples Fox09 and Fox10 (Figure 3 and Table 2), however these samples had air mass source regions away from populated/industrialized land masses (Figure S3a). Together, these results indicate that direct transport (e.g., particle-bound) from Eurasian sources was unimportant for the deposition of PFAS on Foxfonna and that the period of 24 h daylight during April–August is a significant time for the radical-mediated degradation of PFAS precursors in the atmosphere and the subsequent deposition of transformation products in the Arctic.

To understand whether FTOHs could be a possible source of PFCAs to Foxfonna, even:odd molar ratios were calculated for C_2_–C_11_ PFCAs (Table 2), as was done for the Lomonosovfonna ice core previously.^17^ Furthermore, these calculations were expected to contribute to a better understanding of the sources of PFOA to Foxfonna. Surface snow samples, where the PFCA in question was < MQL were not used in the calculation. The ratios of TFA:PFPrA and PFBA:PFPeA were all >3.6, indicating a nonfluorotelomer derived precursor source. This agrees with the Lomonosovfonna ice core record (where their ratios were >2.8). For PFHxA:PFHpA, 50% of the ratios were between 0.5 and 2 (i.e., within a factor of 2). This indicates that 6:2 FTOH could be a possible precursor source to PFHxA and PFHpA. More significant was the ratio for PFDA:PFUnDA where 86% was between 0.5 and 2. This indicates that 10:2 FTOH was a likely precursor source for PFDA and PFUnDA at Foxfonna. This agrees with the findings on Lomonosovfonna (where 82% was between 0.5 and 2). Ratios for PFOA:PFNA were only 33% between 0.5 and 2 indicating that existing knowledge about 8:2 FTOH atmospheric degradation was infrequently observed at Foxfonna.^11^ This is in contrast to what was observed on Lomonosovfonna (82% between 0.5 and 2). Nonetheless those values between 0.5 and 2 at Foxfonna all occurred consecutively during snow sampling in late April–June 2019 (samples Fox07–Fox09). It could be possible that during this time, when 24 h daylight was established, degradation from 8:2 FTOH became the dominant source of PFOA and PFNA to Foxfonna. However, outside of this period (when ratios ranged 2.9–16) an alternative source/atmospheric process was dominant. This could also be from a long-range atmospheric process since the PFOA:PFNA molar ratios in surface snow were also high at the reference sites (2.5–15), including on Lomonosovfonna. Nonetheless PFNA (i.e., the major PFAA degradation product of 8:2 FTOH) deposition during three precipitation events in late April–June (Fox07–Fox09), represented 60% of the total flux of PFNA, respectively, across all 10 samples collected from Foxfonna (Table 2). This suggests that this period during late April–June was a significant time for 8:2 FTOH degradation and that this precursor was likely still the largest contributor to PFNA deposition on Foxfonna, as was found to be the case on the remote ice cap of Lomonosovfonna.

PFAS were also investigated for correlations with Na^+^—a marine aerosol tracer (Table 2). No C_5_–C_11_ PFCA were found to have significant positive correlation with Na^+^ (*r* < 0.54, *p* > 0.11). The same was also observed for HFPO-DA (*r* = 0.04, *p* = 0.91), PFHxS (*r* = 0.41, *p* = 0.24), PFOS (*r* = 0.31, *p* = 0.38) and FBSA (*r* = −0.57, *p* = 0.08). These results are in agreement with the findings of Björnsdotter et al. where Na^+^ was also not found to correlate with TFA, PFPrA, PFBA, and TFMS in surface snow on Foxfonna. This was also the case for remote Arctic ice core records from Lomonosovfonna, Svalbard and the Devon Ice Cap in Arctic Canada.^16,17^ Hence, marine aerosols do not likely provide a long-range atmospheric pathway for the movement of PFAS to the Arctic or within the Arctic. This is an important observation since the Arctic Ocean contains a variety of PFAS,^8^ and it is therefore likely that this is not able to be significantly redistributed to the terrestrial Arctic environment (e.g., ice caps) via marine aerosols.

Differences between PFAS levels and distribution profiles in surface snow from Lomonosovfonna, and the ice core drilled at the same location, suggest that an ice core provides an overview of the atmospheric processes, whereas surface snow samples, only represent the atmospheric conditions with respect to the precipitation event sampled. As such, this study only provides observations from one year of sampling (2019) and longer-term snow sampling would further improve our understanding of PFAS in the Arctic atmosphere. This study has sampled a large area for each precipitation event (on average 1.9 m^2^ at Foxfonna), however variations on a potentially heterogeneous snow surface could contribute to the variations in PFAS fluxes. Re-emission from surface snow back to the atmosphere is known to occur for volatile neutral PFAS.^34^ This study attempted to control for this factor by sampling surface snow as soon as possible after the targeted precipitation event and by consistently sampling only the upper 5 cm of the snow surface. Furthermore, post-deposition degradation of precursors in surface snow is also thought to possibly influence PFAA levels in the days following the precipitation event.^41^ For snow samples collected during complete or partial darkness, additional solar radiation to the snow surface would be limited. However, during summer, incoming solar radiation penetrating the snow surface could also be potentially significant for post-deposition degradations. For example, for Fox09 (collected June 2019), the snow surface was exposed to an additional 3.4 MJ m^–2^ solar flux (over 24 h) between the end of the precipitation event and subsequent snow sampling (calculated from ERA5 reanalysis). However, this is only 2% of the solar flux associated with the airmass parcel linked to the Fox09 precipitation event (185 MJ m^–2^). Although the mechanisms and significance of post-deposition precursor degradations are unclear, seasonal variations in solar radiation could potentially provide an additional energy input for chemical transformations in surface snow during summer months.

This study has been able to collect samples as soon as possible after the precipitation event, whereas some PFAS in the Lomonosovfonna ice core has been subject to >10 years of post-deposition effects, such as meltwater percolation.^17^ Like the ice core from Lomonosovfonna, this study has also been unable to distinguish between particle bound and non-particle bound PFAS in the surface snow. Particle bound transport may be important to the deposition of some PFAS. However, since it is known that the formation of PFAAs from the degradation of precursors is likely a gaseous process,^11,35−38^ it is likely many PFAAs exist as nonparticle bound upon formation. Subsequent gas-particle phase partitioning in the air remains unclear.^53,54^

### Environmental Implications

Existing field evidence for the atmospheric formation of PFCAs has thus far been limited to snow pit and ice core studies, and it has largely relied on homologue ratios between PFCAs to establish a precursor source.^12,16^ This study has taken a different approach by directly linking seasonal atmospheric photochemistry to PFCA formation. This was achieved by measuring the seasonal deposition of PFAAs and comparing this with incoming solar fluxes. Like PFCAs, PFOS also had large seasonal variations in deposition fluxes on Foxfonna. While fluorotelomer derived precursors (e.g., FTOHs) have been established as a significant atmospheric precursor source for PFCAs,^12,16^ no study has yet empirically established an atmospheric precursor source for PFOS. It has been previously suggested that direct particle-bound transport of PFOS might be important to the atmospheric deposition of PFOS in the Arctic.^17^ However, based on FLEXPART atmospheric modeling, results from this study indicated that direct atmospheric transport from source regions (e.g., particle-bound) was not important to the transport of PFOS to the Arctic. Instead, like PFCAs, the deposition of PFOS in this study was linked to seasonal atmospheric photochemistry indicating an atmospheric precursor source for PFOS. This provides the first evidence for the possible atmospheric formation of PFOS from precursors. There is limited existing evidence for the atmospheric formation of PFSAs from precursors, although a simulated atmospheric laboratory study has observed the formation of PFBS from *N*-methyl perfluorobutane sulfonamidoethanol (MeFBSE), by addition of a hydroxyl radical to the sulfonyl bond, followed by cleavage of the S–N bond.^37^ Hence, precursors for PFOS could include C_8_ FASAs and FASEs (e.g., MeFOSE). While PFOS deposition was highest in August, deposition of most C_2_–C_11_ PFCAs were highest during June. These differences could be due to the different reaction pathways required for the atmospheric formation of PFOS compared to PFCAs. PFOS can form from C_8_ FASAs/FASEs precursors via a two-step addition–elimination reaction,^37^ whereas PFCAs form from several sequential reactions (during the radical degradation of precursors, e.g. FTOHs).^11^ Regardless, the structural isomer profile of PFOS in this study, and the ice core from Lomonosovfonna,^17^ confirm that these atmospheric PFOS precursors have been originally manufactured by ECF, as for C_8_ FASAs and FASEs (e.g., EtFOSE).^49^ Measurements of precursors in the Arctic atmosphere show that C_4_ FASAs and FASEs (e.g., MeFBSA and MeFBSE) can be equally, if not more prevalent, than C_8_ FASAs and FASEs.^30,31,34^ These C_4_ precursors could also be an atmospheric source for PFBS, but PFBS detection frequencies remained low on Foxfonna and in the Lomonosovfonna ice core (<25%, Table 1). However, the *N*-dealkylated C_4_ sulfonamide, FBSA, showed high detection frequencies at these locations (≥80%, Table 1). On Foxfonna, its deposition was 190 times higher during 24 h daylight and its significant seasonality in deposition was linked to solar flux (Figure 3 and Table 2). Previously, FBSA was tentatively observed as a product of EtFBSA during a hydroxyl radical initiated degradation whereby successive oxidation of the ethyl chain resulted in the formation of FBSA.^36^ Together these results suggest that, like PFOS, FBSA atmospheric deposition was driven by the atmospheric degradation of precursors. Given the ubiquity of C_4_ and C_8_ FASAs and FASEs in the atmosphere worldwide,^31^ these precursors will likely provide a global source for FBSA and PFOS atmospheric deposition.

This study is the first to detect HFPO-DA (GenX) in Arctic precipitation, including at remote sites. Atmospheric emissions of HFPO-DA have been identified from a fluoropolymer manufacturing facility in the United States and evidence suggested potential for HFPO-DA long-range transport.^55^ In this study, HFPO-DA deposition was linked to seasonal light indicating that a photochemical process was likely enabling its atmospheric formation from a precursor(s) source, as found for PFOS and PFCAs. One possible precursor could be the HFPO-DA equivalent primary alcohol, C_3_F_7_OCF(CF_3_)CH_2_OH (2-perfluoropropoxy-2,3,3,3-tetrafluoropropanol, CAS no. 26537-88-2). During its atmospheric degradation, its terminal alcohol could be oxidized to the carboxylic acid to form HFPO-DA (akin to the aforementioned degradation of 6:2 FTOH to 6:2 FTCA). Fluorinated alcohols of the form F(CF_2_)_*n*_CH_2_OH, where *n* = 1–4, have been found to have atmospheric lifetimes of 164 days with respect to hydroxyl radical degradation.^56^ Hence the chemically similar C_3_F_7_OCF(CF_3_)CH_2_OH could also have a sufficiently long atmospheric lifetime to undergo long-range atmospheric transport to the Arctic, where it could then degrade to HFPO-DA. C_3_F_7_OCF(CF_3_)CH_2_OH is used in the production of perfluoropolyethers (along with a number of other compounds which contain a C_3_F_7_OCF(CF_3_)CF_2_OR substructure, where R = a polyfluorinated chain).^57^ This indicates a possible industrial source for this precursor, although there have been no reports yet of this HFPO-DA precursor in the atmosphere or wider environment. Regardless, this study has found evidence for an atmospheric process which enables HFPO-DA long-range transport to the Arctic, which possibly explains the widespread detection of HFPO-DA in the Arctic Ocean seawater by Joerss et al.^52^

## Supporting Information Available

Full details of the chemicals and reagents, sample extraction, instrument analysis, quality control and quality assurance, long-range atmospheric dispersion modeling (FLEXPART), major ion analysis, and information on the seasonal variations of PFAS in surface snow in Longyearbyen can be found in the Supporting Information (pages S1–S8). Lists of target analytes, abbreviations, CAS numbers, MRM transitions, internal and recovery standards used, LODs and MQLs, extraction efficiencies, and the repeatability of the instrument method can also be found. Tables with PFAS concentrations and fluxes can also be found as well as figures and tables showing the precipitation and timings of each precipitation event (PDF)
