# Supplementary material for: Sources and Seasonal Variations of Per- and Polyfluoroalkyl Substances (PFAS) in Surface Snow in the Arctic
Source: Environ Sci Technol. 2024 Nov 26;58(49):21817–28. doi: 10.1021/acs.est.4c08854 (PMC11636200; doi:10.1021/acs.est.4c08854)
Supplement: Supplementary file 1 — es4c08854_si_001.pdf [file es4c08854_si_001.pdf]

Sources and Seasonal Variations of Per- and Polyfluoroalkyl Substances (PFAS) in Surface Snow in the Arctic – Supporting Information

William F. Hartz,[a,b,c,d*] Maria K. Björnsdotter,[d,e] Leo W. Y. Yeung,[d] Jack D. Humby,[f] Sabine Eckhardt,[c] Nikolaos Evangeliou,[c] Ingrid Ericson Jogsten,[d] Anna Kärrman,[d] Roland Kallenborn[g,h]

[a]Department of Earth Sciences, University of Oxford, South Parks Road, Oxford, OX1 3AN, U.K.

[b]Department of Arctic Geology, University Centre in Svalbard (UNIS), NO-9171, Longyearbyen, Svalbard, Norway

[c]NILU, Instituttveien 18, NO-2007 Kjeller, Norway

[d]Man-Technology-Environment Research Centre (MTM), Örebro University, SE-701 82 Örebro, Sweden

[e]Institute of Environmental Assessment and Water Research (IDAEA-CSIC), C/Jordi Girona, 18-26, 08034 Barcelona, Catalonia, Spain

[f]Ice Dynamics and Paleoclimate, British Antarctic Survey, High Cross, Cambridge, CB3 0ET, United Kingdom

[g]Faculty of Chemistry, Biotechnology and Food Sciences (KBM), Norwegian University of Life Sciences (NMBU), NO-1432 Ås, Norway

[h]University of the Arctic (UArctic), Yliopistonkatu 8, 96300 Rovaniemi, Finland

*Corresponding author, e-mail: whar@nilu.no / william.hartz@oru.se

Number of tables: 17, Number of figures: 3, Number of pages: 29.

Table of Contents

**Chemicals and Reagents**	S1
**Instrument Analysis**	S1
**Quality Assurance and Quality Control**	S2
**Long-range Atmospheric Dispersion Modelling - FLEXPART**	S4
**Ion Chromatography for Quantification of Major Ion Concentrations**	S5
**Seasonal Variations of PFAS in Surface Snow in Longyearbyen**	S7
**References**	S8
**Table S1.** List of target analytes, abbreviations, and CAS numbers	S9
**Table S2.** MRM transitions and, internal and recovery standards used for quantification.	S10
**Table S3.** Instrument limits of quantification, method limits of quantification, extraction efficiencies and repeatability of the instrument method	S11
**Table S4**. The extraction efficiency for those compounds with available equivalent mass-labelled internal and recovery standards	S12
**Table S5**. Further extraction efficiencies for the nine neutral compounds	S12
**Table S6.** MRM transitions, instrument limits of quantification (LOQ), and method limits of quantification (MQL) for branched isomers of PFHxS and PFOS.	S12
**Table S7.** Concentrations (pg L^{-1}) of PFAS at the reference sites	S13
**Table S8.** Concentrations (pg L^{-1}) of PFAS at Foxfonna	S14
**Table S9.** Concentrations (pg L^{-1}) of PFAS at KHO	S15
**Table S10** Concentrations (pg L^{-1}) of PFAS at UNIS, Longyearbyen	S16
**Table S11.** Fluxes (ng m^{-2}) of PFAS at the reference sites	S17
**Table S12.** Fluxes (ng m^{-2}) of PFAS at Foxfonna	S18
**Table S13.** Fluxes (ng m^{-2}) of PFAS at KHO	S19
**Table S14.** Fluxes (ng m^{-2}) of PFAS at UNIS, Longyearbyen	S20
**Table S15.** Percentage of linear isomers, and concentrations of linear and branched isomers of PFOS	S21
**Table S16.** Percentage of linear isomers, and concentrations of linear and branched isomers of PFHxS	S22
**Table S17.** Snow fluxes (kg/m^2), the date and time (UTC, nearest hour) of the onset and termination of each precipitation event, and the date and time of snow sampling.	S23
**Figure S2.** Precipitation data for all precipitation events	S24
**Figure S3.** FLEXPART backward air mass trajectory frequency plots for Fox01 – Fox10	S28

Chemicals and Reagents

Standards of native perfluoroalkyl carboxylic acids (PFCAs; C_4-C_{12}), perfluoroalkyl sulfonic acids (PFSAs; C_3-C_{10} and C_{12} including branched standards) and mass-labelled standards of PFCAs (C_4-C_{12}), PFSAs (C_4, C_6, C_8) were from Wellington laboratories (Guelph, ON, Canada). Perfluoropropanoic acid was from Sigma-Aldrich, (Oakville, ON, Canada). Trifluoromethane sulfonic acid (TFMS) was from Sigma-Aldrich, (Stockholm, Sweden), and potassium salt of perfluoroethane sulfonate was obtained from Kanto Chemical Co., Inc., (Portland, OR, USA). Mass-labelled standard of TFA ($^{13}C_2$-TFA) was purchased from Toronto Research Chemicals Inc, (Toronto, ON, Canada). The purity of all standards was above 97%. Glass microfiber filters (Whatman) and ammonium acetate (≥99.0%) was purchased from Sigma-Aldrich, (Stockholm, Sweden). Glacial acetic acid (EMPROVE® EXPERT, Ph. Eur., JP, USP) was purchased from Merck, (Darmstadt, Germany). Analytical reagent grade ammonia solution, HPLC grade methanol (≥99.8%) and LC-MS grade methanol (≥99.9%) were from Fischer Scientific, (Ottawa, ON, Canada). Weak anion exchange solid-phase extraction (WAX-SPE) cartridges were obtained from Waters Corporation, (Milford, MA, USA).

Instrument Analysis

Both neutral and anionic elutes were evaporated to approximately 0.5 mL at 60 °C and 400 mbar, transferred to an LC vial and then further evaporated to 100 µL under a gentle stream of nitrogen. For quantification of the anionic PFAS, 40 µL of the anionic fraction was transferred to new LC vials and of 10 µL were injected into the ultra-performance liquid chromatography (UPLC) MS/MS system after addition of 60 µL of 2 mM aqueous ammonium acetate. For quantification of neutral PFAS, aliquots of 10 µL of the neutral fraction were injected into the UPLC-MS/MS.

Mass-labelled internal standards and recovery standards (1 ng) were added to the samples prior to filtration and prior to injection, respectively, to monitor the recovery of the method.

Separation and quantification of the neutral PFAS and the remaining anionic PFAS was performed using UPLC-MS/MS (Acquity Ultra Performance Liquid Chromatograph and Xevo TQ-S, Waters Corporation, Milford, MA, USA) operated in negative electrospray ionization mode. A UPLC BEH C18 column (2.1 mm i.d., 100 mm length, 1.7 µm particle size) (Waters Corporation, Milford, MA, USA) maintained at 50 °C was used to achieve chromatographic separation. The mobile phase for UPLC-MS/MS analysis consisted of 2 mM NH_4Ac in MeOH:Milli-Q (3:7) (A) and 2 mM NH_4Ac in MeOH (B) at a flow rate of 0.3 mL/min. The gradient of the mobile phase started with an initial B concentration of 1% for 1 min. This was then increased to 100% over 12 min, held for 1 min, and finally re-conditioned for 3 min. The source parameters were set as following: capillary voltage, 0.7 kV; source temperature, 150 °C; desolvation temperature, 400 °C; cone gas flow, 150 L/h; desolvation gas flow, 800 L/h; collision gas flow, 0.2 mL/min; nebulizer, 6.5 bar. At least two MRM transitions were monitored for each target analyte except MeFOSA and EtFOSA where only one transition was monitored. MRM transitions for all target analytes are provided in Table S2.

Quality Assurance and Quality Control

Linear regression analysis showed good linearity for each analyte ($R^2 > 0.99$) in the range from 2.0 to 100 ng/mL for UPLC and SFC. Instrumental limits of quantification (LOQs) were set as the lowest calibration point with a signal-to-noise ratio of at least ten. For UPLC the repeatability of the instrumental method was evaluated based on repeated injections (n = 8) of a standard with a concentration of 4 ng/mL (or 10 ng/mL for neutral target analytes), the relative standard deviation of repeated injections was in the range 1.0 – 3.7 % for C_5 to C_{11} PFCAs and C_5 – C_{10} PFSAs.

Isotope dilution was used for quantification. For those target analytes that did not have corresponding mass-labelled standards, the homologue closest in retention time was used for quantification (Table S2). Extraction efficiencies of all analytes were assessed based on the peak area of native standards spiked to test samples (n = 3) compared to that of the native standards in solvent after subtraction of the background concentrations in the samples (Table S3). Test samples consisted of 250 mL of melted surface snow from one sample from Foxfonna, spiked with 1000 pg of native standard. The extraction efficiencies for C_5 to C_{11} PFCAs, PFHxS and PFOS were in the range 33 – 69%. The repeatability of the extraction method was evaluated based on the relative standard deviation of spiked test samples (n = 3) at a concentration of 1 ng per 250 mL sample. The relative standard deviation of spiked test samples was in the range 8.7 – 21% for C_5 to C_{11} PFCAs, PFHxS and PFOS. For those analytes with available corresponding mass-labelled internal and recovery standards (C_5 – C_{11} PFCAs, PFHxS and PFOS), their extraction efficiency was also assessed by comparing the peak area of the spiked internal standard and recovery standard across all samples (n = 36). Their extraction efficiencies were in the range 58 – 86% (Table S4), and the relative standard deviation was in the range 10 – 14%. For the 9 neutral analytes, the extraction efficiencies were also assessed by comparing the peak areas of their respective internal and surrogate recovery standards, across all samples (n = 36). Their extraction efficiencies were in the range 3.1 – 42% (Table S5), and the relative standard deviation was in the range 2.0 – 28%. The method limits of quantification (MQLs) were calculated as the average concentration in repeated blank extractions (n = 5) plus three times the standard deviation. For those analytes that were not observed in blank extractions, the instrument LOQ was used as the MQL. Detailed information about the precision of the analytical method, internal and recovery standards, extraction efficiencies and repeatability, and LOQs and MQLs, are provided in Tables S3 – S5.

Prior to sample collection, a high-density polyethylene barrel and aluminum shovel were precleaned with soapy water, followed by rinsing several times with tap water, Milli-Q water rinse five times and then methanol rinse three times. Transport to each sampling site was done by ski or snowmobile. The final approach to the sampling site was done on foot from downwind. Nitrile gloves were worn during snow sampling. The upper 0 – 5 cm of the surface snow was collected into the barrel, which was then sealed and transported back to the University Centre in Svalbard (UNIS), where it was melted in the fridge at 5 °C and bottled into precleaned polypropylene containers, after homogenizing the melted sample by up ending the barrel several times. Polypropylene containers were precleaned with soapy water, followed by rinsing several times with tap water, Milli-Q water rinse five times, and then a rinse with acetone followed by methanol. It was then rinsed three times with the melted snow sample from the barrel, prior to filling the container. Subsamples from the barrel were also taken for major ion analysis. The bottled and melted snow samples for PFAS analysis were then stored in the fridge at 2 – 4 °C until they were transported cold to Örebro University, Sweden where they were stored at 8 °C until extraction.

A field blank was included to ensure that no contamination occurred during surface snow sampling. The field blank comprised of Milli-Q water in a sample container that was brought to the field and kept opened for the duration of sampling. The container was then resealed and transported to the laboratory where it was treated in the same way as the samples. None of the target analytes in the field blank were detected >MQL.

Long-range Atmospheric Dispersion Modelling - FLEXPART

The Lagrangian Particle Dispersion Model FLEXPART version 10.4 was used to investigate the air mass origin of the deposited PFAS.[1] The model was driven with ERA5 hourly reanalysis meteorological wind fields from the European Centre for Medium-Range Weather Forecasts

(ECMWF) consisting of 137 vertical levels and a horizontal resolution of 0.5°×0.5°.[2] The emission sensitivities were calculated in backward mode, using FLEXPART's feature that reconstructs wet and dry deposition with backward simulations,[3] an extension of the traditional backward simulation for atmospheric concentrations.[4] PFAS were tracked backwards for 10 days. This was based on the expected atmospheric lifetime of PFOA.[5]

For the reconstruction of wet deposition, computational particles were released at altitudes 0 – 20 km at the receptor and exact time of sample collection, as scavenging can occur at any height of the atmosphere depending on the location of clouds and precipitation. For dry deposition, particles were released at 0 – 30 m at the respective receptors, as this shallow layer is equal to the height of the layer in which, in forward mode, particles are subject to dry deposition. The resulting footprint emission sensitivities (FES) show the probability of any gridded region to emit PFAS species that could be transported and deposited at the receptor and is nothing more than the source-receptor relationship between emissions and deposition for a 10 day tracking. ERA5 solar downward UV-radiation at the surface (given in J/m^2) over the whole model column (0 – 30 km) throughout the 10-day tracking are combined with the FES (in m) that resulted for the duration of the snow events. The resulting quantity (J/m^2) is the solar radiation flux normalized with the FES and shows the exposure to UV radiation for the photochemically produced PFAS degradation products along the calculated trajectories. Except for dry and wet deposition,[6] FLEXPART accounts for turbulent, unresolved mesoscale motions,[7,8] and convection.[9]

Ion Chromatography for Quantification of Major Ion Concentrations

A 50 mL subsample for major ion analysis was collected from the melted snow sample and stored in a fridge. Cation and anion concentrations were measured using Dionex ICS-4000 Integrion anion and cation systems. A Dionex AS-AP autosampler was used to supply sample to each

instrument. Calibration was achieved using a range of calibration standards. All calibration standards were prepared from purchased (1000 ppm) Sigma Aldrich (U.K.) standards by a series of gravimetric dilutions.

The cation instrument used a Dionex Ionpac CG16-4μm (2 x 50 mm) guard column and CS16 (4 μm, 2 x 250 mm) analytical column. Samples were loaded into a 250 µL sample loop. A 32 – 42 mM methane sulfonic acid (MSA) multistep eluent gradient was used for effective separation of the analytes. The eluent was produced using a Dionex eluent generator cartridge (EGC 500 KOH) and was pumped through the columns at a flow rate of 0.20 mL min^{-1} at a pressure of 3300 psi for a 25 min chromatogram. The eluent ions were removed from the column effluent using a cation electrolytically regenerated suppressor (Dionex CERS 500), before conductivity detection of the effluent.

The anion system used a Dionex Ionpac AG17-C (2 μm, 2 x 50 mm) guard column and AS17-C (2 x 250 mm) analytical column. Samples were loaded to a 250 µL sample loop. A 1 – 40 mM potassium hydroxide multistep eluent gradient was used for effective separation of the analytes. The eluent was produced using a Dionex eluent generator cartridge (EGC 500 KOH) and was pumped through the columns at a flow rate of 0.29 mL min^{-1} at a pressure of 2300 psi for a 20 min chromatogram. The eluent ions were removed from the column effluent using an anion electrolytically regenerated suppressor (Dionex AERS 500), before conductivity detection of the eluate.

Seasonal variations of PFAS in Surface Snow in Longyearbyen

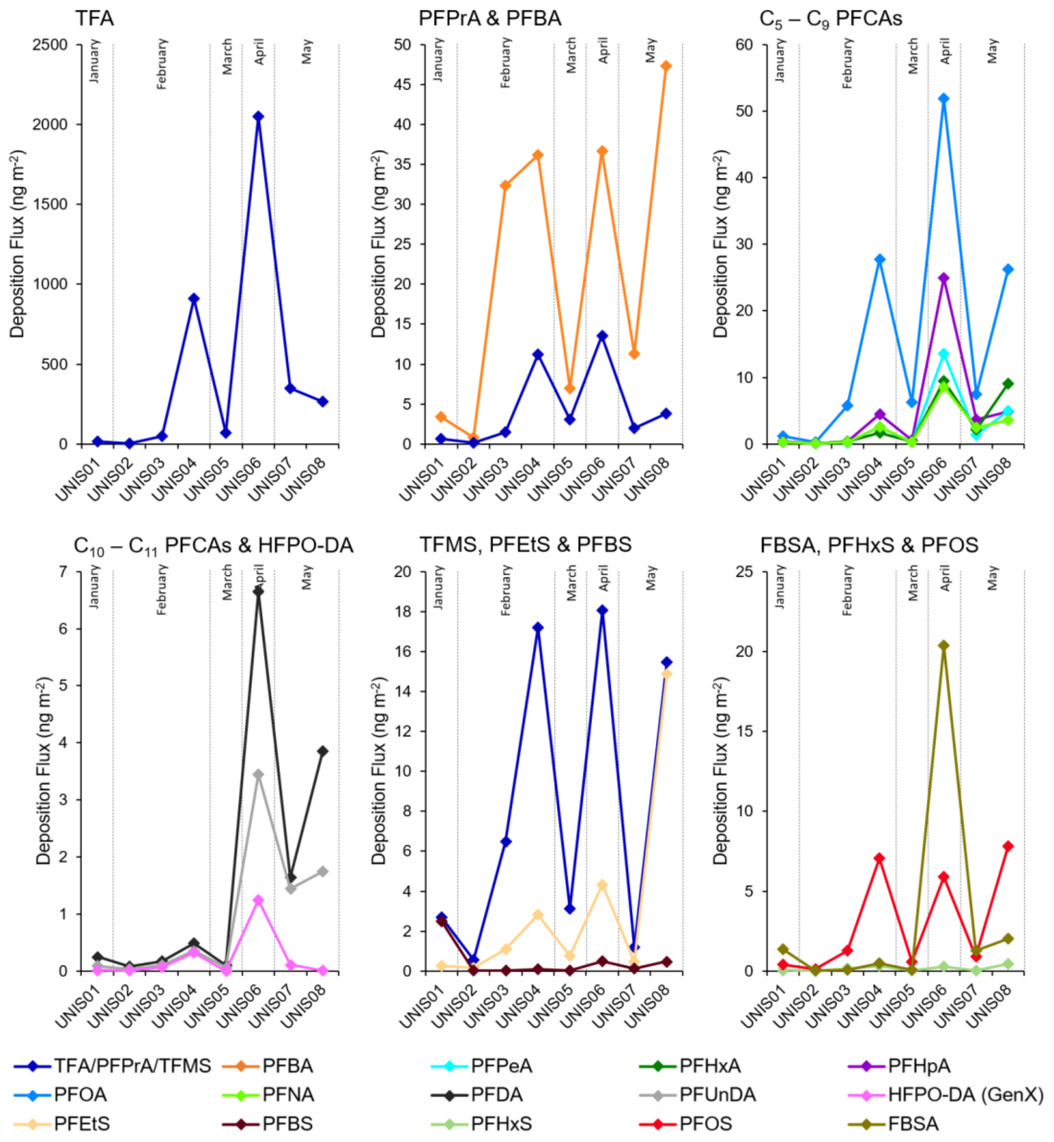

Figure S1: Deposition fluxes per precipitation event (ng m^{-2}) of TFMS, PFEtS, PFBS, PFHxS, PFOS, FBSA, HFPO-DA (GenX) and C_2 – C_{11} PFCAs in surface snow outside UNIS in Longyearbyen during January – May 2019.

As discussed in *Comparison between Snow Sampling Locations and the Lomonosovfonna 2019 Ice Core*, surface snow at UNIS in Longyearbyen has likely been influenced by local sources of C_4 – C_{11} PFCAs, PFEtS, PFBS, PFHxS and PFOS. This confirms existing knowledge that human

activities contribute to the PFAS input in the Arctic.[10] Investigating the fluxes of PFAS in surface snow sampled January – May 2019 outside UNIS in Longyearbyen offered the opportunity to inspect for seasonal variations in PFAS in surface snow as a result of seasonal variations of human activities in the settlement.

Fluxes of most PFAS were low during January and parts of February. After this period, PFAS fluxes in the surface snow were highly variable, likely as a result of several local factors. Higher fluxes might occur due to (i) sporadic increases in PFAS usage in Longyearbyen, (ii) increased emission from local sources due to variations in snow cover during the sampling period and (iii) increased degradation in the atmosphere/snow surface from local sources of precursors after 24-hour daylight started on 19th April 2019. PFAS fluxes might also be occasionally altered as a result of sporadic temperature increases in Longyearbyen, initiating meltwater percolation in the surface snow.

References

1. Pisso, I. *et al.* The Lagrangian particle dispersion model FLEXPART version 10.4. *Geosci. Model Dev.* **12**, 4955–4997 (2019).
2. Hersbach, H. *et al.* The ERA5 global reanalysis. *Q. J. R. Meteorol. Soc.* **146**, 1999–2049 (2020).
3. Eckhardt, S. *et al.* Source-receptor matrix calculation for deposited mass with the Lagrangian particle dispersion model FLEXPART v10.2 in backward mode. *Geosci. Model Dev.* **10**, 4605–4618 (2017).
4. Seibert, P. & Frank, A. Source-receptor matrix calculation with a Lagrangian particle dispersion model in backward mode. *Atmos. Chem. Phys.* **4**, 51–63 (2004).
5. Hurley, M. D. *et al.* Atmospheric chemistry of perfluorinated carboxylic acids: Reaction with OH radicals and atmospheric lifetimes. *J. Phys. Chem. A* **108**, 615–620 (2004).
6. Grythe, H. *et al.* A new aerosol wet removal scheme for the Lagrangian particle model FLEXPART v10. *Geosci. Model Dev.* **10**, 1447–1466 (2017).
7. Cassiani, M., Stohl, A. & Brioude, J. Lagrangian Stochastic Modelling of Dispersion in the Convective Boundary Layer with Skewed Turbulence Conditions and a Vertical Density Gradient: Formulation and Implementation in the FLEXPART Model. *Boundary-Layer Meteorol.* **154**, 367–390 (2015).
8. Stohl, A., Forster, C., Frank, A., Seibert, P. & Wotawa, G. Technical note: The Lagrangian particle dispersion model FLEXPART version 6.2. *Atmos. Chem. Phys.* **5**, 2461–2474 (2005).
9. Forster, C., Stohl, A. & Seibert, P. Parameterization of Convective Transport in a Lagrangian Particle Dispersion Model and Its Evaluation. *J. Appl. Meteorol. Climatol.* **46**, 403–422 (2007).
10. Ali, A. M. *et al.* The fate of poly- And perfluoroalkyl substances in a marine food web influenced by land-based sources in the Norwegian Arctic. *Environ. Sci. Process. Impacts* **23**, 588–604 (2021).
11. Björnsdotter, M. K. *et al.* Levels and Seasonal Trends of C1-C4 Perfluoroalkyl Acids and the Discovery of Trifluoromethane Sulfonic Acid in Surface Snow in the Arctic. *Environ. Sci. Technol.* **55**, 15853–15861 (2021).

Table S1. List of target analytes, abbreviations, and CAS numbers

Abbreviation	Target analyte	CAS	Intrument Method
TFA	Trifluoroacetic acid	76-05-1	SFC
PFPrA	Perfluoropropanoic acid	422-64-0	SFC
PFBA	Perfluorobutanoic acid	375-22-4	SFC
PFPeA	Perfluoropentanoic acid	2706-90-3	UPLC
PFHxA	Perfluorohexanoic acid	307-24-4	UPLC
PFHpA	Perfluoroheptanoic acid	375-85-9	UPLC
PFOA	Perfluorooctanoic acid	335-67-1	UPLC
PFNA	Perfluorononanoic acid	375-95-1	UPLC
PFDA	Perfluorodecanoic acid	335-76-2	UPLC
PFUnDA	Perfluoroundecanoic acid	2058-94-8	UPLC
PFDoDA	Perfluorododecanoic acid	307-55-1	UPLC
PFTrDA	Perfluorotridecanoic acid	72629-94-8	UPLC
PFTeDA	Perfluorotetradecanoic acid	376-06-7	UPLC
PFHxDA	Perfluorohexadecanoic acid	67905-19-5	UPLC
PFOcDA	Perfluorooctadecanoic acid	16517-11-6	UPLC
TFMS	Trifluoromethane sulfonic acid	1493-13-6	SFC
PFEtS	Perfluoroethane sulfonic acid	354-88-1	SFC
PFPrS	Perfluoropropane sulfonic acid	423-41-6	SFC
PFBS	Perfluorobutane sulfonic acid	375-73-5	SFC
PFPeS	Perfluoropentane sulfonic acid	2706-91-4	UPLC
PFHxS	Perfluorohexane sulfonic acid	355-46-4	UPLC
PFHpS	Perfluoroheptane sulfonic acid	375-92-8	UPLC
PFOS	Perfluorooctane sulfonic acid	1763-23-1	UPLC
PFNS	Perfluorononane sulfonic acid	68259-12-1	UPLC
PFDS	Perfluorodecane sulfonic acid	335-77-3	UPLC
PFDoDS	Perfluorododecane sulfonic acid	79780-39-5	UPLC
4:2 FTSA	4:2 Fluorotelomer sulfonic acid	757124-72-4	UPLC
6:2 FTSA	6:2 Fluorotelomer sulfonic acid	27619-97-2	UPLC
8:2 FTSA	8:2 Fluorotelomer sulfonic acid	39108-34-4	UPLC
6:2 FTUCA	6:2 Fluorotelomer unsaturated carboxylic acid	70887-88-6	UPLC
8:2 FTUCA	8:2 Fluorotelomer unsaturated carboxylic acid	70887-84-2	UPLC
PFECHS	Perfluoroethylcyclohexane sulfonic acid	72441-89-5	UPLC
6:2 Cl-PFESA	6:2 Chlorinated polyfluorinated ether sulfonate	756426-58-1	UPLC
8:2 Cl-PFESA	8:2 Chlorinated polyfluorinated ether sulfonate	763051-92-9	UPLC
HFPO-DA	Hexafluoropropylene oxide dimer acid	13252-13-6	UPLC
ADONA	3H-perfluoro-3-[(3-methoxy-propoxy)propanoic acid]	919005-14-4	UPLC
FBSA	Perfluorobutane sulfonamide	30334-69-1	UPLC
MeFBSA	Methyl perfluorobutane sulfonamide	68298-12-4	UPLC
FHxSA	Perfluorohexane sulfonamide	41997-13-1	UPLC
MeFHxSA	Methyl perfluorohexane sulfonamide	68259-15-4	UPLC
FOSA	Perfluorooctane sulfonamide	754-91-6	UPLC
MeFOSA	N-Methyl perfluorooctane sulfonamide	31506-32-8	UPLC
EtFOSA	N-Ethyl perfluorooctane sulfonamide	4151-50-2	UPLC
MeFOSE	N-Methyl perfluorooctane sulfonamidoethanol	24448-09-7	UPLC
EtFOSE	N-Ethyl perfluorooctane sulfonamidoethanol	1691-99-2	UPLC

Table S2. MRM transitions and internal standards used for quantification.

Abbreviation	1st MRM transition	2nd MRM transition	Internal standard	Recovery Standard
TFA	112.90 > 68.96	-	^{13}C-M2TFA	
PFPrA	162.97 > 118.90	-	^{13}C-M4PFBA	
PFBA	212.97 > 169.00	-	^{13}C-M4PFBA	^{13}C-M3PFBA
PFPeA	262.97 > 219.00	-	^{13}C-M3PFPeA	^{13}C-M5PFPeA
PFHxA	312.97 > 269.00	312.97 > 118.95	^{13}C-M2PFHxA	^{13}C-M5PFHxA
PFHpA	362.97 > 319.00	362.97 > 168.97	^{13}C-M4PFHpA	^{13}C-M8PFOA
PFOA	412.97 > 369.00	412.97 > 168.97	^{13}C-M4PFOA	^{13}C-M8PFOA
PFNA	462.99 > 419.00	462.99 > 219.00	^{13}C-M5PFNA	^{13}C-M9PFNA
PFDA	512.97 > 469.00	512.97 > 219.00	^{13}C-M2PFDA	^{13}C-M6PFDA
PFUnDA	562.97 > 268.99	562.97 > 519.00	^{13}C-M2PFUnDA	^{13}C-M7PFUnDA
PFDoDA	612.97 > 569.00	612.97 > 168.96	^{13}C-M2PFDoDA	
PFTrDA	662.90 > 619.00	662.90 > 168.96	^{13}C-M2PFDoDA	
PFTeDA	712.90 > 669.00	712.90 > 168.97	^{13}C-M2PFTeDA	
PFHxDA	812.90 > 769.00	812.90 > 168.96	^{13}C-M2PFHxDA	
PFOcDA	912.90 > 869.00	912.90 > 168.96	^{13}C-M2PFHxDA	
TFMS	149.12 > 79.91	149.12 > 98.95	^{13}C-M3PFBS	
PFEtS	198.80 > 79.80	198.80 > 98.90	^{13}C-M3PFBS	
PFPrS	248.90 > 79.90	248.90 > 98.90	^{13}C-M3PFBS	
PFBS	298.90 > 79.96	298.90 > 98.90	^{13}C-M3PFBS	
PFPeS	348.90 > 79.96	348.90 > 98.96	^{18}O-M2PFHxS	
PFHxS	398.90 > 79.96	398.90 > 98.90<br>398.90 > 119.01<br>398.90 > 319.00	^{18}O-M2PFHxS	^{13}C-M3PFHxS
PFHpS	448.97 > 79.96	448.97 > 98.90	^{13}C-M4PFOS	
PFOS	498.97 > 79.96	498.97 > 98.96<br>498.97 > 169.03<br>498.97 > 419.00	^{13}C-M4PFOS	^{13}C-M8PFOS
PFNS	548.90 > 79.96	548.90 > 98.96	^{13}C-M4PFOS	
PFDS	598.97 > 79.96	598.97 > 98.90	^{13}C-M4PFOS	
PFDoDS	698.90 > 79.96	698.90 > 98.90	^{13}C-M4PFOS	
4:2 FTSA	327.00 > 307.00	329.00 > 81.00	^{13}C-M2-6:2 FTSA	
6:2 FTSA	427.00 > 407.00	427.00 > 81.00	^{13}C-M2-6:2 FTSA	
8:2 FTSA	527.00 > 507.00	527.00 > 80.00	^{13}C-M2-8:2 FTSA	
6:2 FTUCA	356.90 > 292.91	356.9 > 242.95	^{13}C-M2-6:2 FTUCA	
8:2 FTUCA	456.90 > 392.94	456.90 > 342.88	^{13}C-M2-8:2 FTUCA	
PFECHS	460.84 > 380.942	460.84 > 98.882	^{13}C-M4PFOA	
6:2 Cl-PFESA	530.904 > 350.98	530.904 > 98.95	^{13}C-M4PFOS	
8:2 Cl-PFESA	630.904 > 450.98	630.904 > 98.95	^{13}C-M4PFOS	
HFPO-DA	284.90 > 168.70	284.9 > 184.80	^{13}C-M3HFPO-DA	
ADONA	376.97 > 250.83	376.97 > 84.69	^{13}C-M3HFPO-DA	
FBSA	297.90 > 77.92	297.90 > 118.94	^{13}C-M8FOSA	
MeFBSA	311.97 > 218.95	311.97 > 111.93	^{13}C-M8FOSA	
FHxSA	397.9 > 77.92	397.90 > 168.94	^{13}C-M8FOSA	
MeFHxSA	411.97 > 168.93	411.97 > 318.96	^{13}C-M8FOSA	
FOSA	497.90 > 78.00	497.90 > 168.90	^{13}C-M8FOSA	
Me-FOSA	512.00 > 169.00	-	^{13}C-d3-N-MeFOSA	
EtFOSA	526.00 > 169.00	-	^{13}C-d5-N-EtFOSA	
MeFOSE	616.00 > 59.00	556.03 > 121.99	^{13}C-d7-N-MeFOSE	
EtFOSE	630.00 > 59.00	570.096 > 135.983	^{13}C-d9-N-EtFOSE	

Table S3. Instrument limits of quantification (LOQ) (pg L^{-1}) and method limits of quantification (MQL) (pg L^{-1}). Extraction efficiencies (%) and repeatability (%) is based spiked test samples (*n* = 3). Repeatability (%) of the instrument method was based on repeated standard injections (*n* = 8 for UPLC, *n* = 10 for SFC)

**Analyte**	**LOQ (pg/L)**	**MQL (pg/L)**	**Extraction efficiency (%)**	**Extraction method repeatability (%)**	**Instrument method repeatability (%)**
TFA[a]	9.1	9.1	121 ± 5	2.7	4.1
PFPrA[a]	9.1	9.1	67 ± 8	11	2.4
PFBA[a]	9.1	58.2	71 ± 6	14	3.0
PFPeA	2.3	5.9	69 ± 15	4.3	1.2
PFHxA	2.3	5.2	65 ± 10	4.9	1.2
PFHpA	2.3	4.3	63 ± 14	8.9	2.0
PFOA	2.3	65.2	57 ± 21	15	1.0
PFNA	2.3	4.1	46 ± 14	27	1.4
PFDA	2.3	15.5	40 ± 8.7	26	2.5
PFUnDA	2.3	6.9	33 ± 11	27	3.7
PFDoDA	2.3	2.6	28 ± 14	49	1.9
PFTrDA	2.3	1.7	31 ± 18	44	3.7
PFTeDA	2.3	2.3	46 ± 29	49	4.7
PFHxDA	2.3	7.9	53 ± 23	50	3.7
PFOcDA	2.3	3.1	85 ± 88	110	25
TFMS[a]	9.1	9.1	80 ± 16	15	2.8
PFEtS[a]	9.1	45	81 ± 9	2.2	0.33
PFPrS[a]	9.1	9.1	83 ± 8	6.0	2.4
PFBS[a]	2.3	12.9	80 ± 12	3.3	1.7
PFPeS	2.3	2.3	69 ± 10	9.2	1.6
PFHxS	2.3	2.7	58 ± 11	17	1.2
PFHpS	9.1	9.1	48 ± 11	21	2.7
PFOS	2.3	7.9	42 ± 9.1	15	2.1
PFNS	9.1	9.1	30 ± 5.8	18	2.8
PFDS	2.3	2.3	24 ± 10	39	1.5
PFDoDS	2.3	2.3	27 ± 16	38	2.1
4:2 FTSA	2.3	2.3	160 ± 42	13	3.1
6:2 FTSA	2.3	8.4	180 ± 89	50	5.0
8:2 FTSA	2.3	2.3	78 ± 14	29	5.0
6:2 FTUCA	2.3	2.3	10 ± 4.0	42	1.8
8:2 FTUCA	2.3	2.3	2.1 ± 1.2	60	2.3
PFECHS	2.3	2.3	49 ± 11	21	1.4
6:2 Cl-PFESA	2.3	2.3	37 ± 8.6	28	1.6
8:2 Cl-PFESA	2.3	2.3	26 ± 12	46	1.6
HFPO-DA	2.3	2.3	50 ± 10	11	2.5
ADONA	2.3	2.3	53 ± 7.2	13	1.5
FBSA	2.3	2.3	38 ± 20	40	6.8
MeFBSA	9.1	9.1	12 ± 8.6	73	12
FHxSA	2.3	2.3	29 ± 13	44	8.6
MeFHxSA	9.1	9.1	4.9 ± 4.5	91	7.7
FOSA	2.3	3.6	23 ± 7.8	31	7.9
MeFOSA	22.7	22.7	6.6 ± 6.0	91	12
EtFOSA	22.7	22.7	6.4 ± 5.1	69	13
MeFOSE	2.3	16.7	13 ± 2.6	35	12
EtFOSE	2.3	11.9	14 ± 4.0	40	12

[a]Previously reported by Björnsdotter et al.[11]

Table S4. The extraction efficiency for those compounds with available equivalent mass-labelled internal and recovery standards, expressed as the average extraction efficiency ± standard deviation of spiked internal standards (*n* = 36)

Analyte	Internal standard	Recovery standard	Extraction efficiency (%)
PFBA	^{13}C-M4PFBA	^{13}C-M3PFBA	69 ± 12
PFPeA	^{13}C-M3PFPeA	^{13}C-M5PFPeA	70 ± 11
PFHxA	^{13}C-M2PFHxA	^{13}C-M5PFHxA	72 ± 12
PFHpA	^{13}C-M4PFHpA	^{13}C-M8PFOA	70 ± 13
PFOA	^{13}C-M4PFOA	^{13}C-M8PFOA	71 ± 11
PFNA	^{13}C-M5PFNA	^{13}C-M9PFNA	65 ± 12
PFDA	^{13}C-M2PFDA	^{13}C-M6PFDA	58 ± 13
PFUnDA	^{13}C-M2PFUnDA	^{13}C-M7PFUnDA	54 ± 14
PFHxS	^{18}O-M2PFHxS	^{13}C-M3PFHxS	86 ± 10
PFOS	^{13}C-M4PFOS	^{13}C-M8PFOS	65 ± 11

Table S5. The extraction efficiency for the nine neutral compounds, expressed as the average extraction efficiency ± standard deviation of spiked internal standards (*n* = 36)

Analyte	Internal standard	Recovery standard	Extraction efficiency (%)
FOSA	^{13}C-M8FOSA[a]	^{13}C-M8PFOS	42 ± 28
MeFOSA	^{13}C-d3-N-MeFOSA	^{13}C-M8PFOS	4.3 ± 2.6
EtFOSA	^{13}C-d5-N-EtFOSA	^{13}C-M8PFOS	3.1 ± 2.0
MeFOSE	^{13}C-d7-N-MeFOSE	^{13}C-M8PFOS	27 ± 19
EtFOSE	^{13}C-d9-N-EtFOSE	^{13}C-M8PFOS	16 ± 12

[a] ^{13}C-M8FOSA is also the internal standard for FBSA, MeFBSA, FHxSA and MeFHxSA

Table S6. MRM transitions, instrument limits of quantification (LOQ) (pg L^{-1}), and method limits of quantification (MQL) (pg L^{-1}) for branched isomers of PFHxS and PFOS.

Abbreviation	1st MRM transition	2nd MRM transition	LOQ (pg L^{-1})	MQL (pg L^{-1})
2/3/4-PFHxS	398.9 > 79.96	398.9 > 98.96	2.3	2.3
1-PFHxS	398.9 > 319.00	398.9 > 98.96	2.3	2.3
3/4/5-PFOS	498.97 > 79.96	498.97 > 98.96	2.3	2.3
6/2-PFOS	498.97 > 169.03	498.97 > 79.96	2.3	2.3
1-PFOS	498.97 > 419.00	498.97 > 169.03	2.3	2.3

Table S7. Concentrations (pg L^{-1}) of PFAS at the reference sites from February – April 2019

	**Drønbreen**	**Lomonosovfonna01**	**Lomonosovfonna02**	**Nordmannsfonna**
PFPeA	61.5	27.7	57.3	162
PFHxA	55.5	45.5	59.6	124
PFHpA	89.9	34.9	115	477
PFOA	260	305	243	370
PFNA	35.3	22.1	71.7	166
PFDA	<MQL	<MQL	<MQL	26.3
PFUnDA	7.54	6.94	18.0	35.7
PFDoDA	4.13	3.26	<MQL	8.65
PFTrDA	3.11	<MQL	3.07	8.28
PFTDA	<MQL	2.83	2.28	3.28
PFHxDA	<MQL	<MQL	<MQL	<MQL
PFOcDA	<MQL	<MQL	<MQL	<MQL
PFPeS	<MQL	<MQL	<MQL	<MQL
PFHxS	11.5	7.7	7.39	10.6
PFHpS	<MQL	<MQL	<MQL	<MQL
PFOS	287	128	251	136
PFNS	<MQL	<MQL	<MQL	<MQL
PFDS	<MQL	<MQL	<MQL	<MQL
PFDoDS	<MQL	<MQL	<MQL	<MQL
4:2 FTSA	<MQL	<MQL	<MQL	<MQL
6:2 FTSA	19.9	13.3	13.5	16.9
8:2 FTSA	<MQL	<MQL	<MQL	<MQL
6:2 FTUCA	66.8	22.8	76.9	40
8:2 FTUCA	31.7	<MQL	54.1	<MQL
PFECHS	<MQL	<MQL	<MQL	<MQL
6:2 Cl-PFESA	<MQL	<MQL	<MQL	<MQL
8:2 Cl-PFESA	<MQL	<MQL	<MQL	<MQL
HFPO-DA	<MQL	3.26	5.72	7.74
ADONA	<MQL	<MQL	<MQL	<MQL
FBSA	5.58	3.59	<MQL	8.15
MeFBSA	11	52.1	31.6	18.3
FHxSA	27.5	18.5	9.43	33.1
MeFHxSA	<MQL	<MQL	<MQL	<MQL
FOSA	4.52	4.44	<MQL	4.96
MeFOSA	<MQL	<MQL	<MQL	<MQL
EtFOSA	<MQL	<MQL	<MQL	<MQL
MeFOSE	18.9	<MQL	17.1	20.4
EtFOSE	<MQL	<MQL	<MQL	<MQL

Table S8. Concentrations (pg L^{-1}) of PFAS at Foxfonna from January – August 2019

	**Fox01**	**Fox02**	**Fox03**	**Fox04**	**Fox05**	**Fox06**	**Fox07**	**Fox08**	**Fox09**	**Fox10**
PFPeA	6.04	45.0	35.2	22.9	55.7	54.1	231	453	290	49.9
PFHxA	23.6	58.1	50.6	35.3	80.0	67.5	326	540	265	69.6
PFHpA	9.63	72.3	31.3	33.5	64.8	88.9	1350	1790	974	787
PFOA	336	494	422	334	241	248	640	563	704	327
PFNA	<MQL	41.8	28.7	23	40.9	38.4	449	836	449	128
PFDA	<MQL	19.7	<MQL	<MQL	16.7	16.9	72.0	80.3	56.5	17.8
PFUnDA	<MQL	8.01	<MQL	<MQL	17.7	18.2	75.0	109	72.1	19.3
PFDoDA	3.15	<MQL	2.63	<MQL	<MQL	6.47	16	14.4	<MQL	<MQL
PFTrDA	<MQL	1.95	1.96	1.85	5.17	9.36	19.1	12.7	10.1	2.85
PFTDA	<MQL	<MQL	<MQL	<MQL	<MQL	2.8	5.64	5.24	2.28	<MQL
PFHxDA	<MQL	<MQL	<MQL	<MQL	<MQL	<MQL	<MQL	<MQL	<MQL	<MQL
PFOcDA	<MQL	<MQL	<MQL	<MQL	<MQL	<MQL	<MQL	<MQL	<MQL	<MQL
PFPeS	<MQL	<MQL	<MQL	<MQL	<MQL	<MQL	<MQL	<MQL	<MQL	<MQL
PFHxS	4.47	14.4	19.1	10.2	7.01	5.26	23.1	7.42	10.8	8.39
PFHpS	<MQL	<MQL	<MQL	<MQL	<MQL	<MQL	<MQL	<MQL	<MQL	<MQL
PFOS	61.9	113	214	88.7	159	107	331	177	258	137
PFNS	<MQL	<MQL	<MQL	<MQL	<MQL	<MQL	<MQL	<MQL	<MQL	<MQL
PFDS	<MQL	<MQL	<MQL	<MQL	<MQL	<MQL	<MQL	<MQL	<MQL	<MQL
PFDoDS	<MQL	<MQL	<MQL	<MQL	<MQL	<MQL	<MQL	<MQL	<MQL	<MQL
4:2 FTSA	<MQL	<MQL	<MQL	<MQL	<MQL	<MQL	<MQL	<MQL	<MQL	<MQL
6:2 FTSA	11.2	13.9	13.5	13.1	15.4	15	40.8	24.1	28.3	17.3
8:2 FTSA	<MQL	<MQL	<MQL	<MQL	<MQL	<MQL	<MQL	2.66	<MQL	<MQL
6:2 FTUCA	6.44	17.8	37.8	64.4	13.8	47.3	<MQL	31.4	<MQL	51.6
8:2 FTUCA	<MQL	<MQL	<MQL	<MQL	<MQL	<MQL	<MQL	<MQL	<MQL	<MQL
PFECHS	<MQL	<MQL	<MQL	<MQL	<MQL	<MQL	<MQL	<MQL	<MQL	<MQL
6:2 Cl-PFESA	<MQL	<MQL	<MQL	<MQL	<MQL	<MQL	<MQL	<MQL	<MQL	<MQL
8:2 Cl-PFESA	<MQL	<MQL	<MQL	<MQL	<MQL	<MQL	<MQL	<MQL	<MQL	<MQL
HFPO-DA	<MQL	5.04	6.72	3.75	16.2	25.9	24.4	60.5	22.4	9.23
ADONA	<MQL	<MQL	<MQL	<MQL	<MQL	<MQL	3.19	<MQL	<MQL	<MQL
FBSA	2.63	<MQL	<MQL	2.52	6.4	183	99.9	192	76.3	57.6
MeFBSA	<MQL	<MQL	<MQL	<MQL	13.3	51.3	31.8	<MQL	43.0	19.4
FHxSA	16.5	21.2	12.8	5.65	6.05	9.02	29.5	13.5	19.3	41.3
MeFHxSA	<MQL	<MQL	<MQL	<MQL	<MQL	<MQL	<MQL	<MQL	<MQL	<MQL
FOSA	14.6	12.4	<MQL	20.6	4.6	4.01	5.4	15.3	46.2	13.2
MeFOSA	<MQL	<MQL	<MQL	<MQL	<MQL	<MQL	<MQL	<MQL	<MQL	<MQL
EtFOSA	<MQL	128	<MQL	<MQL	<MQL	<MQL	<MQL	<MQL	<MQL	<MQL
MeFOSE	<MQL	103	<MQL	<MQL	21.8	16.9	24.3	<MQL	<MQL	<MQL
EtFOSE	13.4	247	<MQL	17.7	13.0	<MQL	<MQL	<MQL	<MQL	<MQL

Table S9. Concentrations (pg L^{-1}) of PFAS at KHO from January – June 2019

	**KHO01**	**KHO02**	**KHO03**	**KHO04**	**KHO05**	**KHO06**	**KHO07**	**KHO08**	**KHO09**
PFPeA	45.7	57.9	20.9	13.0	38.5	31.6	169	141	233
PFHxA	45.8	36.5	32.6	34.1	30.5	39.4	90.8	224	234
PFHpA	75.3	119	99.6	19.9	91.3	32.5	706	1080	902
PFOA	1330	533	114	487	325	323	444	509	514
PFNA	42.4	114	35.1	18.8	42.8	13.4	173	176	364
PFDA	18.2	<MQL	<MQL	<MQL	<MQL	<MQL	30.4	48.1	46.5
PFUnDA	8.13	10.4	12.3	<MQL	7.91	<MQL	26.4	36.7	58.4
PFDoDA	4.04	2.82	3.58	2.75	<MQL	<MQL	5.83	13.4	11.0
PFTrDA	2.8	2.68	5.09	<MQL	2.43	2.19	5.5	16.7	16.1
PFTDA	<MQL	2.42	<MQL	<MQL	<MQL	<MQL	3.23	5.66	5.12
PFHxDA	<MQL	<MQL	<MQL	<MQL	<MQL	<MQL	<MQL	<MQL	<MQL
PFOcDA	<MQL	<MQL	<MQL	<MQL	<MQL	<MQL	<MQL	<MQL	<MQL
PFPeS	<MQL	3.96	<MQL	<MQL	<MQL	<MQL	<MQL	<MQL	<MQL
PFHxS	16.1	43.2	6.9	13.8	9.9	6.65	7.26	8.78	7.52
PFHpS	<MQL	<MQL	<MQL	<MQL	<MQL	<MQL	<MQL	<MQL	<MQL
PFOS	172	312	64.2	142	60.9	71.6	55.8	95.4	79.5
PFNS	<MQL	<MQL	<MQL	<MQL	<MQL	<MQL	<MQL	<MQL	<MQL
PFDS	<MQL	<MQL	<MQL	<MQL	<MQL	<MQL	<MQL	<MQL	<MQL
PFDoDS	<MQL	<MQL	<MQL	<MQL	<MQL	<MQL	<MQL	<MQL	<MQL
4:2 FTSA	<MQL	<MQL	<MQL	<MQL	<MQL	<MQL	<MQL	<MQL	<MQL
6:2 FTSA	10.8	14.4	9.38	12.5	12.1	11.5	13.8	16.1	15.8
8:2 FTSA	<MQL	<MQL	<MQL	<MQL	<MQL	<MQL	<MQL	<MQL	<MQL
6:2 FTUCA	<MQL	8.75	<MQL	6.87	19.2	8.14	2.70	38.6	5.51
8:2 FTUCA	<MQL	<MQL	<MQL	<MQL	<MQL	<MQL	3.32	<MQL	17.7
PFECHS	<MQL	10.7	<MQL	<MQL	<MQL	<MQL	<MQL	<MQL	<MQL
6:2 Cl-PFESA	<MQL	<MQL	<MQL	<MQL	<MQL	<MQL	<MQL	<MQL	<MQL
8:2 Cl-PFESA	<MQL	<MQL	<MQL	<MQL	<MQL	<MQL	<MQL	<MQL	<MQL
HFPO-DA	<MQL	14.0	<MQL	7.14	3.89	7.44	22.5	19.7	25.5
ADONA	<MQL	<MQL	<MQL	<MQL	<MQL	<MQL	<MQL	<MQL	4.05
FBSA	3.91	4.71	<MQL	3.06	2.74	134	26.9	52.2	25.3
MeFBSA	10.0	18.5	<MQL	<MQL	<MQL	<MQL	<MQL	<MQL	<MQL
FHxSA	24.7	40.1	12.9	6.47	3.89	10.7	13.6	10.3	9.27
MeFHxSA	<MQL	<MQL	<MQL	<MQL	<MQL	<MQL	<MQL	<MQL	<MQL
FOSA	43.7	11.4	4.25	<MQL	3.71	3.94	10.8	6.01	8.00
MeFOSA	<MQL	<MQL	<MQL	<MQL	<MQL	<MQL	<MQL	<MQL	<MQL
EtFOSA	28.8	<MQL	<MQL	<MQL	<MQL	<MQL	<MQL	<MQL	<MQL
MeFOSE	53.5	<MQL	<MQL	<MQL	<MQL	<MQL	22.3	<MQL	27.1
EtFOSE	178	<MQL	<MQL	<MQL	<MQL	12.3	16.2	<MQL	14.8

Table S10. Concentrations (pg L^{-1}) of PFAS at UNIS, Longyearbyen from January – May 2019

	**UNIS01**	**UNIS02**	**UNIS03**	**UNIS04**	**UNIS05**	**UNIS06**	**UNIS07**	**UNIS08**
PFPeA	253	183	75.1	196	105	749	787	932
PFHxA	319	229	123	130	138	523	1210	1690
PFHpA	384	401	106	338	211	1370	2020	917
PFOA	1180	1390	1850	2120	2400	2860	4120	4900
PFNA	274	402	100	196	111	470	1390	667
PFDA	244	359	55.8	37.2	39.4	366	904	720
PFUnDA	93.6	147	32.6	27.0	24.4	190	799	325
PFDoDA	48.4	81.5	16.3	<MQL	<MQL	124	1320	417
PFTrDA	24.5	50.3	7.25	5.86	3.26	62.2	567	206
PFTDA	20.8	23.8	8.67	9.19	6.17	51.6	1580	380
PFHxDA	12.5	8.75	<MQL	13.0	9.18	24.4	771	272
PFOcDA	11.0	<MQL	<MQL	28.4	<MQL	<MQL	280	142
PFPeS	<MQL	<MQL	9.28	<MQL	<MQL	<MQL	<MQL	<MQL
PFHxS	52.2	74.7	52.7	24.5	20.3	16.5	23.7	81.9
PFHpS	<MQL	13.8	<MQL	23.1	<MQL	<MQL	<MQL	20.5
PFOS	405	463	411	541	228	324	497	1460
PFNS	<MQL	<MQL	<MQL	<MQL	<MQL	<MQL	<MQL	<MQL
PFDS	<MQL	<MQL	<MQL	<MQL	<MQL	<MQL	<MQL	<MQL
PFDoDS	<MQL	<MQL	<MQL	<MQL	<MQL	<MQL	<MQL	<MQL
4:2 FTSA	<MQL	<MQL	<MQL	<MQL	<MQL	<MQL	<MQL	<MQL
6:2 FTSA	20.4	17.5	28.6	26.2	25.2	40.2	117	149
8:2 FTSA	<MQL	<MQL	<MQL	<MQL	11.6	<MQL	<MQL	21.8
6:2 FTUCA	<MQL	<MQL	133	45.9	83.8	<MQL	<MQL	<MQL
8:2 FTUCA	<MQL	<MQL	<MQL	16.2	55.9	<MQL	<MQL	<MQL
PFECHS	15.3	25.6	5.26	6.43	2.92	<MQL	8.39	<MQL
6:2 Cl-PFESA	<MQL	<MQL	<MQL	<MQL	<MQL	<MQL	<MQL	<MQL
8:2 Cl-PFESA	<MQL	<MQL	<MQL	<MQL	<MQL	<MQL	<MQL	<MQL
HFPO-DA	16.0	26.9	22.8	24.6	<MQL	68.7	60.9	<MQL
ADONA	<MQL	<MQL	<MQL	<MQL	<MQL	<MQL	<MQL	<MQL
FBSA	1350	116	22.2	38.7	30.5	1120	714	381
MeFBSA	92.9	73.8	74.2	18.2	29.3	21.6	46.1	48.2
FHxSA	125	83.9	40.4	16.7	14.3	132	117	184
MeFHxSA	<MQL	<MQL	<MQL	<MQL	<MQL	<MQL	<MQL	<MQL
FOSA	31.5	14.2	16.4	8.16	11.9	17.8	156	23.3
MeFOSA	<MQL	<MQL	<MQL	<MQL	<MQL	<MQL	<MQL	<MQL
EtFOSA	<MQL	<MQL	<MQL	<MQL	<MQL	<MQL	<MQL	<MQL
MeFOSE	<MQL	<MQL	<MQL	26.2	35.9	<MQL	<MQL	68.2
EtFOSE	<MQL	<MQL	501	131	132	58.8	431	<MQL

Table S11. Fluxes (pg m^{-2}) of PFAS at the reference sites from February – April 2019

	**Drønbreen**	**Lomonosovfonna01**	**Lomonosovfonna02**	**Nordmannsfonna**
TFA	8500	700000	120000	62000
PFPrA	260	8500	2200	1100
PFBA	380	7100	2700	1200
PFPeA	40	1800	170	120
PFHxA	36	1400	280	120
PFHpA	59	5400	220	240
PFOA	170	4200	1900	500
PFNA	23	1900	140	150
PFDA	<MQL	300	<MQL	<MQL
PFUnDA	4.9	400	43	37
PFDoDA	2.7	98	20	<MQL
PFTrDA	2.0	94	<MQL	6.4
PFTDA	<MQL	37	18	4.7
PFHxDA	<MQL	<MQL	<MQL	<MQL
PFOcDA	<MQL	<MQL	<MQL	<MQL
TFMS	750	17000	4500	1800
PFEtS	<MQL	<MQL	<MQL	<MQL
PFPrS	<MQL	<MQL	<MQL	<MQL
PFBS	<MQL	<MQL	<MQL	<MQL
PFPeS	<MQL	<MQL	<MQL	<MQL
PFHxS	7.5	120	48	15
PFHpS	<MQL	<MQL	<MQL	<MQL
PFOS	190	1500	800	520
PFNS	<MQL	<MQL	<MQL	<MQL
PFDS	<MQL	<MQL	<MQL	<MQL
PFDoDS	<MQL	<MQL	<MQL	<MQL
4:2 FTSA	<MQL	<MQL	<MQL	<MQL
6:2 FTSA	13	190	83	28
8:2 FTSA	<MQL	<MQL	<MQL	<MQL
6:2 FTUCA	44	450	140	160
8:2 FTUCA	21	<MQL	<MQL	110
PFECHS	<MQL	<MQL	<MQL	<MQL
6:2 Cl-PFESA	<MQL	<MQL	<MQL	<MQL
8:2 Cl-PFESA	<MQL	<MQL	<MQL	<MQL
HFPO-DA	<MQL	88	20	12
ADONA	<MQL	<MQL	<MQL	<MQL
FBSA	3.7	92	22	<MQL
MeFBSA	7.2	210	320	66
FHxSA	18	370	120	20
MeFHxSA	<MQL	<MQL	<MQL	<MQL
FOSA	3.0	56	28	<MQL
MeFOSA	<MQL	<MQL	<MQL	<MQL
EtFOSA	<MQL	<MQL	<MQL	<MQL
MeFOSE	12	230	<MQL	35
EtFOSE	<MQL	<MQL	<MQL	<MQL

Table S12. Fluxes (pg m^{-2}) of PFAS at Foxfonna from January – August 2019

	Fox01	Fox02	Fox03	Fox04	Fox05	Fox06	Fox07	Fox08	Fox09	Fox10
TFA	7000	8200	26000	12000	21000	920000	390000	150000	1700000	830000
PFPrA	300	230	1100	310	810	9800	2400	1300	9300	8700
PFBA	<MQL	690	1200	320	1900	10000	3700	1600	5200	8400
PFPeA	5.8	29	130	17	110	930	540	400	1800	850
PFHxA	23	37	180	27	160	1200	760	470	1700	1200
PFHpA	9.2	46	110	25	130	1500	3100	1600	6100	13000
PFOA	320	320	1500	250	480	4200	1500	500	4400	5600
PFNA	<MQL	27	100	17	81	660	1000	740	2800	2200
PFDA	<MQL	13	<MQL	<MQL	33	290	170	71	350	300
PFUnDA	<MQL	5.1	<MQL	<MQL	35	310	180	96	450	330
PFDoDA	3.0	<MQL	9.5	<MQL	<MQL	110	37	13	<MQL	<MQL
PFTrDA	<MQL	1.2	7.1	1.4	10	160	45	11	63	48
PFTDA	<MQL	<MQL	<MQL	<MQL	<MQL	48	13	4.6	14	<MQL
PFHxDA	<MQL	<MQL	<MQL	<MQL	<MQL	<MQL	<MQL	<MQL	<MQL	<MQL
PFOcDA	<MQL	<MQL	<MQL	<MQL	<MQL	<MQL	<MQL	<MQL	<MQL	<MQL
TFMS	1200	3200	3300	780	1100	11000	850	260	10000	23000
PFEtS	<MQL	<MQL	<MQL	<MQL	<MQL	<MQL	<MQL	<MQL	<MQL	<MQL
PFPrS	<MQL	<MQL	<MQL	<MQL	<MQL	<MQL	<MQL	<MQL	<MQL	<MQL
PFBS	<MQL	<MQL	<MQL	<MQL	<MQL	<MQL	31	<MQL	<MQL	<MQL
PFPeS	<MQL	<MQL	<MQL	<MQL	<MQL	<MQL	<MQL	<MQL	<MQL	<MQL
PFHxS	4.3	9.2	69	7.7	14	90	54	6.5	67	140
PFHpS	<MQL	<MQL	<MQL	<MQL	<MQL	<MQL	<MQL	<MQL	<MQL	<MQL
PFOS	59	72	780	67	320	1800	770	160	1600	2300
PFNS	<MQL	<MQL	<MQL	<MQL	<MQL	<MQL	<MQL	<MQL	<MQL	<MQL
PFDS	<MQL	<MQL	<MQL	<MQL	<MQL	<MQL	<MQL	<MQL	<MQL	<MQL
PFDoDS	<MQL	<MQL	<MQL	<MQL	<MQL	<MQL	<MQL	<MQL	<MQL	<MQL
4:2 FTSA	<MQL	<MQL	<MQL	<MQL	<MQL	<MQL	<MQL	<MQL	<MQL	<MQL
6:2 FTSA	11	8.9	49	9.9	31	260	95	21	180	290
8:2 FTSA	<MQL	<MQL	<MQL	<MQL	<MQL	<MQL	<MQL	2.3	<MQL	<MQL
6:2 FTUCA	6.1	11	140	49	27	810	<MQL	28	<MQL	880
8:2 FTUCA	<MQL	<MQL	<MQL	<MQL	<MQL	<MQL	<MQL	<MQL	<MQL	<MQL
PFECHS	<MQL	<MQL	<MQL	<MQL	<MQL	<MQL	<MQL	<MQL	<MQL	<MQL
6:2 Cl-PFESA	<MQL	<MQL	<MQL	<MQL	<MQL	<MQL	<MQL	<MQL	<MQL	<MQL
8:2 Cl-PFESA	<MQL	<MQL	<MQL	<MQL	<MQL	<MQL	<MQL	<MQL	<MQL	<MQL
HFPO-DA	<MQL	3.2	24	2.8	32	440	57	53	140	160
ADONA	<MQL	<MQL	<MQL	<MQL	<MQL	<MQL	7.5	<MQL	<MQL	<MQL
FBSA	2.5	<MQL	<MQL	1.9	13	3100	230	170	470	980
MeFBSA	<MQL	<MQL	<MQL	<MQL	26	880	74	<MQL	270	330
FHxSA	16	14	46	4.3	12	150	69	12	120	700
MeFHxSA	<MQL	<MQL	<MQL	<MQL	<MQL	<MQL	<MQL	<MQL	<MQL	<MQL
FOSA	14	7.9	<MQL	16	9.1	69	13	13	290	220
MeFOSA	<MQL	<MQL	<MQL	<MQL	<MQL	<MQL	<MQL	<MQL	<MQL	<MQL
EtFOSA	<MQL	82	<MQL	<MQL	<MQL	<MQL	<MQL	<MQL	<MQL	<MQL
MeFOSE	<MQL	66	<MQL	<MQL	43	290	57	<MQL	<MQL	<MQL
EtFOSE	13	160	<MQL	13	26	<MQL	<MQL	<MQL	<MQL	<MQL

Table S13. Fluxes (pg m^{-2}) of PFAS at KHO from January – June 2019

	KHO01	KHO02	KHO03	KHO04	KHO05	KHO06	KHO07	KHO08	KHO09
TFA	5700	11000	27000	180000	41000	1400000	220000	75000	400000
PFPrA	210	390	1100	4200	1100	9600	2200	770	5000
PFBA	580	780	330	5200	820	11000	1200	720	5800
PFPeA	47	42	72	190	92	550	360	110	1300
PFHxA	47	27	110	500	73	690	190	180	1300
PFHpA	77	87	350	290	220	570	1500	870	5000
PFOA	1400	390	400	7200	780	5700	950	410	2900
PFNA	43	83	120	280	100	230	370	140	2000
PFDA	19	<MQL	<MQL	<MQL	<MQL	<MQL	65	39	260
PFUnDA	8.3	7.6	43	<MQL	19	<MQL	56	30	330
PFDoDA	4.1	2.1	12	41	<MQL	<MQL	12	11	61
PFTrDA	2.9	2.0	18	<MQL	5.8	38	12	13	90
PFTDA	<MQL	1.8	<MQL	<MQL	<MQL	<MQL	6.9	4.6	29
PFHxDA	<MQL	<MQL	<MQL	<MQL	<MQL	<MQL	<MQL	<MQL	<MQL
PFOcDA	<MQL	<MQL	<MQL	<MQL	<MQL	<MQL	<MQL	<MQL	<MQL
TFMS	1100	970	10000	28000	2300	8700	310	170	1200
PFEtS	100	84	<MQL	<MQL	<MQL	<MQL	<MQL	<MQL	<MQL
PFPrS	<MQL	<MQL	<MQL	<MQL	<MQL	<MQL	<MQL	<MQL	<MQL
PFBS	<MQL	<MQL	<MQL	<MQL	<MQL	<MQL	<MQL	<MQL	<MQL
PFPeS	<MQL	2.9	<MQL	<MQL	<MQL	<MQL	<MQL	<MQL	<MQL
PFHxS	16	31	24	200	24	120	16	7.1	42
PFHpS	<MQL	<MQL	<MQL	<MQL	<MQL	<MQL	<MQL	<MQL	<MQL
PFOS	180	230	220	2100	150	1300	120	77	440
PFNS	<MQL	<MQL	<MQL	<MQL	<MQL	<MQL	<MQL	<MQL	<MQL
PFDS	<MQL	<MQL	<MQL	<MQL	<MQL	<MQL	<MQL	<MQL	<MQL
PFDoDS	<MQL	<MQL	<MQL	<MQL	<MQL	<MQL	<MQL	<MQL	<MQL
4:2 FTSA	<MQL	<MQL	<MQL	<MQL	<MQL	<MQL	<MQL	<MQL	<MQL
6:2 FTSA	11	11	33	180	29	200	30	13	88
8:2 FTSA	<MQL	<MQL	<MQL	<MQL	<MQL	<MQL	<MQL	<MQL	<MQL
6:2 FTUCA	<MQL	6.4	<MQL	100	46	140	5.8	31	31
8:2 FTUCA	<MQL	<MQL	<MQL	<MQL	<MQL	<MQL	7.1	<MQL	99
PFECHS	<MQL	7.8	<MQL	<MQL	<MQL	<MQL	<MQL	<MQL	<MQL
6:2 Cl-PFESA	<MQL	<MQL	<MQL	<MQL	<MQL	<MQL	<MQL	<MQL	<MQL
8:2 Cl-PFESA	<MQL	<MQL	<MQL	<MQL	<MQL	<MQL	<MQL	<MQL	<MQL
HFPO-DA	<MQL	10	<MQL	110	9.3	130	48	16	140
ADONA	<MQL	<MQL	<MQL	<MQL	<MQL	<MQL	<MQL	<MQL	23
FBSA	4.0	3.4	<MQL	45	6.6	2300	58	42	140
MeFBSA	10	14	<MQL	<MQL	<MQL	<MQL	<MQL	<MQL	<MQL
FHxSA	25	29	45	96	9.3	190	29	8.3	52
MeFHxSA	<MQL	<MQL	<MQL	<MQL	<MQL	<MQL	<MQL	<MQL	<MQL
FOSA	45	8.3	15	<MQL	8.9	69	23	4.8	45
MeFOSA	<MQL	<MQL	<MQL	<MQL	<MQL	<MQL	<MQL	<MQL	<MQL
EtFOSA	29	<MQL	<MQL	<MQL	<MQL	<MQL	<MQL	<MQL	<MQL
MeFOSE	55	<MQL	<MQL	<MQL	<MQL	<MQL	48	<MQL	150
EtFOSE	180	<MQL	<MQL	<MQL	<MQL	220	35	<MQL	82

Table S14. Fluxes (pg m^{-2}) of PFAS at UNIS, Longyearbyen from January – May 2019

	**UNIS01**	**UNIS02**	**UNIS03**	**UNIS04**	**UNIS05**	**UNIS06**	**UNIS07**	**UNIS08**
TFA	18000	5600	50000	910000	69000	2100000	350000	270000
PFPrA	670	170	1400	11000	3100	14000	2000	3800
PFBA	3400	820	32000	36000	7000	37000	11000	47000
PFPeA	260	42	230	2600	270	14000	1400	5000
PFHxA	330	52	380	1700	360	9500	2200	9000
PFHpA	390	92	330	4400	550	25000	3700	4900
PFOA	1200	320	5800	28000	6200	52000	7500	26000
PFNA	280	92	310	2600	290	8500	2500	3600
PFDA	250	82	170	490	100	6600	1600	3900
PFUnDA	96	34	100	350	63	3400	1400	1700
PFDoDA	49	19	51	<MQL	<MQL	2300	2400	2200
PFTrDA	25	12	23	77	8.5	1100	1000	1100
PFTDA	21	5.5	27	120	16	940	2900	2000
PFHxDA	13	2.0	<MQL	170	24	440	1400	1500
PFOcDA	11	<MQL	<MQL	370	<MQL	<MQL	510	760
TFMS	2700	560	6500	17000	3100	18000	1200	15000
PFEtS	250	160	1100	2800	750	4300	470	15000
PFPrS	<MQL	<MQL	<MQL	<MQL	<MQL	<MQL	<MQL	<MQL
PFBS	2500	24	<MQL	<MQL	<MQL	480	120	480
PFPeS	<MQL	<MQL	29	<MQL	<MQL	<MQL	<MQL	<MQL
PFHxS	53	17	160	320	53	300	43	440
PFHpS	<MQL	3.2	<MQL	300	<MQL	<MQL	<MQL	110
PFOS	410	110	1300	7100	590	5900	900	7800
PFNS	<MQL	<MQL	<MQL	<MQL	<MQL	<MQL	<MQL	<MQL
PFDS	<MQL	<MQL	<MQL	<MQL	<MQL	<MQL	<MQL	<MQL
PFDoDS	<MQL	<MQL	<MQL	<MQL	<MQL	<MQL	<MQL	<MQL
4:2 FTSA	<MQL	<MQL	<MQL	<MQL	<MQL	<MQL	<MQL	<MQL
6:2 FTSA	21	4.0	89	340	66	730	210	800
8:2 FTSA	<MQL	<MQL	<MQL	<MQL	30	<MQL	<MQL	120
6:2 FTUCA	<MQL	<MQL	410	600	220	<MQL	<MQL	<MQL
8:2 FTUCA	<MQL	<MQL	<MQL	210	150	<MQL	<MQL	<MQL
PFECHS	16	5.9	16	84	7.6	<MQL	15	<MQL
6:2 Cl-PFESA	<MQL	<MQL	<MQL	<MQL	<MQL	<MQL	<MQL	<MQL
8:2 Cl-PFESA	<MQL	<MQL	<MQL	<MQL	<MQL	<MQL	<MQL	<MQL
HFPO-DA	16	6.2	71	320	<MQL	1200	110	<MQL
ADONA	<MQL	<MQL	<MQL	<MQL	<MQL	<MQL	<MQL	<MQL
FBSA	1400	27	69	510	79	20000	1300	2000
MeFBSA	95	17	230	240	76	390	84	260
FHxSA	130	19	130	220	37	2400	210	990
MeFHxSA	<MQL	<MQL	<MQL	<MQL	<MQL	<MQL	<MQL	<MQL
FOSA	32	3.2	51	110	31	320	280	120
MeFOSA	<MQL	<MQL	<MQL	<MQL	<MQL	<MQL	<MQL	<MQL
EtFOSA	<MQL	<MQL	<MQL	<MQL	<MQL	<MQL	<MQL	<MQL
MeFOSE	<MQL	<MQL	<MQL	340	93	<MQL	<MQL	370
EtFOSE	<MQL	<MQL	1600	1700	340	1100	780	<MQL

Table S15. Percentage of linear isomers and concentrations (pg L^{-1}) of linear and perfluoromethyl branched isomers of PFOS. 1-PFOS was <MQL for all subsections.

Surface Snow Sample	3/4/5-PFOS	6/2-PFOS	Linear-PFOS	Linear-PFOS (%)
UNIS01	53.5	34.9	405	82%
UNIS02	61.1	61.5	463	79%
UNIS03	54.1	46.3	411	80%
UNIS04	49.2	42.8	541	85%
UNIS05	25.3	27.8	228	81%
UNIS06	26.2	26.3	324	86%
UNIS07	25.0	36.4	497	89%
UNIS08	144	173	1460	82%
KHO01	19.6	16.3	172	83%
KHO02	52.0	42.6	312	77%
KHO03	9.91	11.9	64.2	75%
KHO04	17.6	15.3	142	81%
KHO05	11.5	8.17	60.9	76%
KHO06	8.58	4.86	71.6	84%
KHO07	9.49	6.97	55.8	77%
KHO08	14.1	16.5	95.4	76%
KHO09	9.66	10.9	79.5	79%
Fox01	7.75	7.22	61.9	81%
Fox02	16.2	16.2	113	78%
Fox03	33.9	23.8	214	79%
Fox04	13.5	10.2	88.7	79%
Fox05	18.5	9.55	159	85%
Fox06	<MQL	11.0	107	91%
Fox07	47.1	38.6	331	79%
Fox08	18.4	17.4	177	83%
Fox09	27.1	26.5	258	83%
Fox10	19.3	13.9	137	81%
Drønbreen	30.3	30.8	287	82%
Lomonosovfonna01	96.7	64.3	339	80%
Lomonosovfonna02	16.9	15.9	136	82%
Nordmannsfonna	31.0	23.0	251	81%

Table S16. Percentage of linear isomers and concentrations (pg L^{-1}) of linear and perfluoromethyl branched isomers of PFHxS. 1-PFHxS was <MQL for all subsections.

Surface Snow Sample	2/3/4-PFHxS	Linear-PFHxS	Linear-PFHxS (%)
UNIS01	<MQL	52.2	100%
UNIS02	<MQL	74.7	100%
UNIS03	<MQL	52.7	100%
UNIS04	<MQL	24.5	100%
UNIS05	<MQL	20.3	100%
UNIS06	<MQL	16.5	100%
UNIS07	<MQL	23.7	100%
UNIS08	<MQL	81.9	100%
KHO01	<MQL	16.1	100%
KHO02	<MQL	43.2	100%
KHO03	<MQL	6.90	100%
KHO04	3.99	13.8	78%
KHO05	<MQL	9.90	100%
KHO06	<MQL	6.65	100%
KHO07	<MQL	7.26	100%
KHO08	<MQL	8.78	100%
KHO09	<MQL	7.52	100%
Fox01	<MQL	4.47	100%
Fox02	<MQL	14.4	100%
Fox03	<MQL	19.1	100%
Fox04	<MQL	10.2	100%
Fox05	<MQL	7.01	100%
Fox06	<MQL	5.26	100%
Fox07	<MQL	23.1	100%
Fox08	<MQL	7.42	100%
Fox09	<MQL	10.8	100%
Fox10	<MQL	8.39	100%
Drønbreen	<MQL	11.5	100%
Lomonosovfonna01	<MQL	53.7	100%
Lomonosovfonna02	<MQL	10.6	100%
Nordmannsfonna	<MQL	7.39	100%

Table S17. Precipitation (kg/m^2), the date and time (UTC, nearest hour) of the onset and termination of each precipitation event and the date and time of snow sampling. Solar fluxes (MJ/m^2) are given for Fox01 – Fox10.

Surface Snow Sample	Precipitation (kg/m^2)	Precipitation Onset Time	Precipitation End Time	Sampling Time	Solar Flux (MJ/m^2)
UNIS01	1.021	03:00 16/01/2019	02:00 18/01/2019	15:00 24/01/2019	-
UNIS02	0.229	03:00 02/02/2019	07:00 03/02/2019	12:00 04/02/2019	-
UNIS03	3.124	21:00 10/02/2019	20:00 12/02/2019	20:00 12/02/2019	-
UNIS04	13.085	08:00 21/02/2019	15:00 24/02/2019	15:00 24/02/2019	-
UNIS05	2.598	03:00 09/03/2019	22:00 11/03/2019	22:00 11/03/2019	-
UNIS06	18.149	10:00 20/04/2019	13:00 21/04/2019	15:00 23/04/2019	-
UNIS07	1.811	18:00 28/04/2019	00:00 30/04/2019	12:00 06/05/2019	-
UNIS08	5.355	08:00 14/05/2019	09:00 16/05/2019	20:00 21/05/2019	-
KHO01	1.020	03:00 16/01/2019	13:00 18/01/2019	13:00 21/01/2019	-
KHO02	0.729	22:00 01/02/2019	11:00 03/02/2019	13:00 04/02/2019	-
KHO03	3.471	19:00 10/02/2019	15:00 12/02/2019	15:00 12/02/2019	-
KHO04	14.767	09:00 21/02/2019	12:00 24/02/2019	12:00 24/02/2019	-
KHO05	2.399	06:00 09/03/2019	15:00 11/03/2019	15:00 11/03/2019	-
KHO06	17.506	10:00 20/04/2019	15:00 21/04/2019	10:00 23/04/2019	-
KHO07	2.140	18:00 28/04/2019	00:00 29/04/2019	15:00 30/04/2019	-
KHO08	0.806	07:00 18/05/2019	06:00 20/05/2019	15:00 20/05/2019	-
KHO09	5.567	00:00 05/06/2019	10:00 09/06/2019	08:00 10/06/2019	-
Fox01	0.954	06:00 16/01/2019	13:00 18/01/2019	11:00 21/01/2019	1.11
Fox02	0.641	13:00 05/02/2019	11:00 07/02/2019	11:00 07/02/2019	23.7
Fox03	3.626	19:00 10/02/2019	00:00 12/02/2019	10:00 14/02/2019	1.89
Fox04	0.754	13:00 26/02/2019	07:00 27/02/2019	17:00 05/03/2019	0.358
Fox05	1.985	08:00 09/03/2019	06:00 11/03/2019	18:00 11/03/2019	1.95
Fox06	17.131	10:00 20/04/2019	15:00 21/04/2019	12:00 23/04/2019	101
Fox07	2.336	18:00 28/04/2019	00:00 29/04/2019	13:00 30/04/2019	75.4
Fox08	0.880	07:00 18/05/2019	06:00 20/05/2019	18:00 20/05/2019	40.7
Fox09	6.224	00:00 05/06/2019	10:00 09/06/2019	10:00 10/06/2019	185
Fox10	16.990	05:00 20/08/2019	16:00 21/08/2019	18:00 21/08/2019	70.4
Drønbreen	0.656	13:00 26/02/2019	03:00 27/02/2019	15:00 28/02/2019	-
Lomonosovfonna01	11.313	17:00 27/03/2019	15:00 30/03/2019	14:00 03/04/2019	-
Lomonosovfonna02	6.216	15:00 04/04/2019	12:00 07/04/2019	12:00 07/04/2019	-
Nordmannsfonna	2.075	15:00 11/04/2019	21:00 12/04/2019	21:00 12/04/2019	-

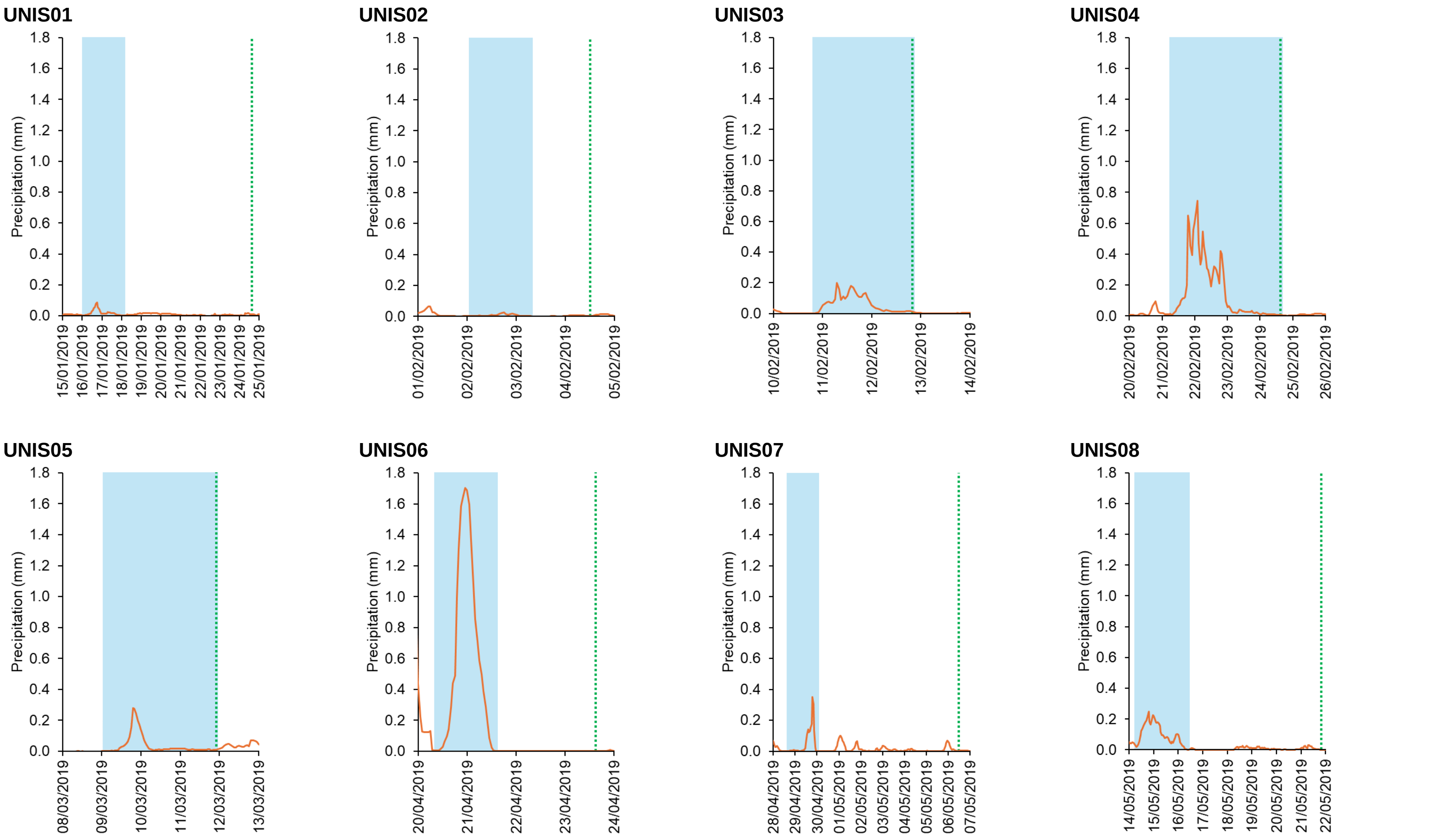

Figure S2a: Meteorological data at UNIS. Orange line = Hourly precipitation from ERA5 (mm). Blue highlight = Duration of precipitation event. Green vertical line = Snow sampling time

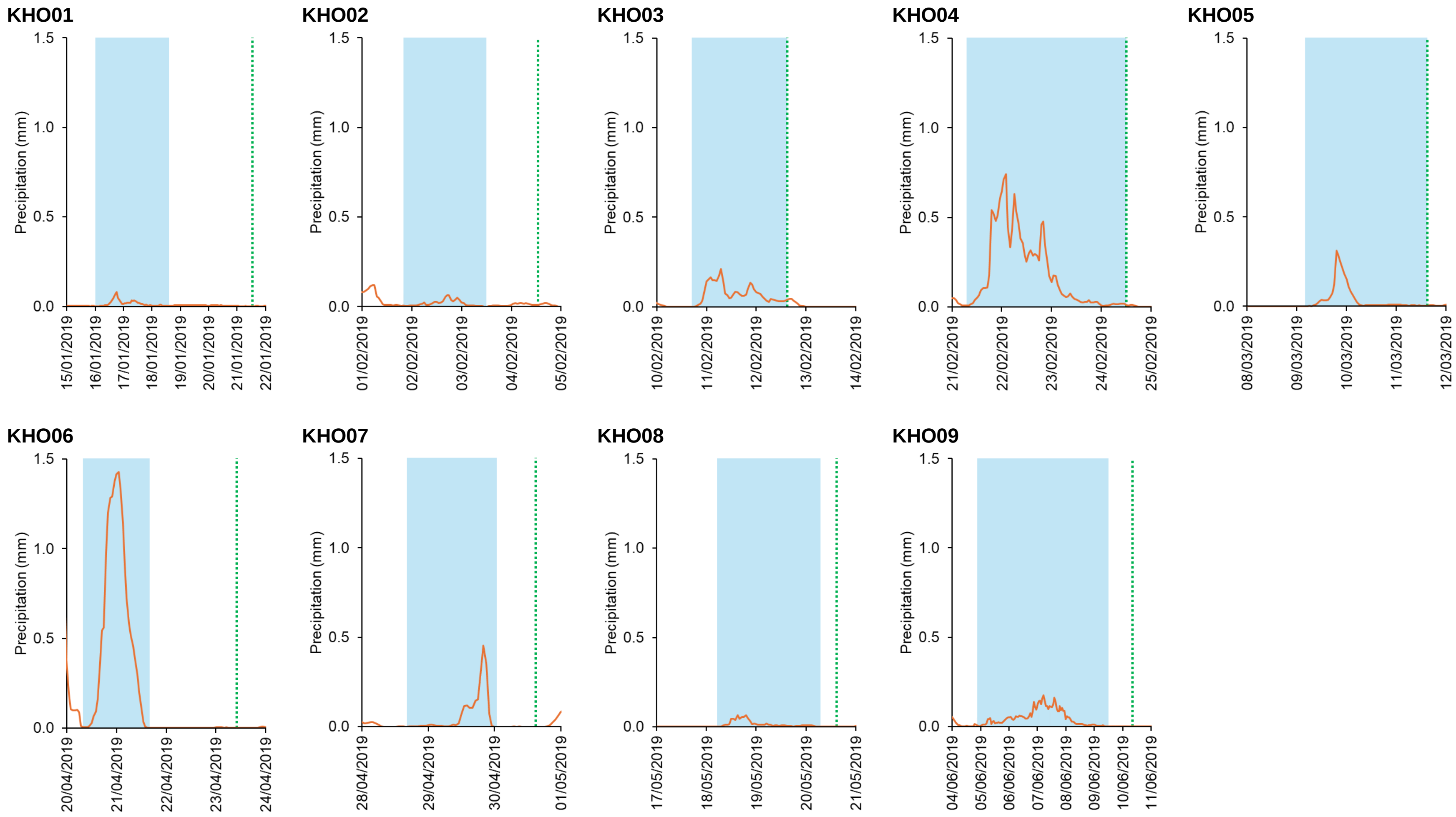

Figure S2b: Meteorological data for KHO. Orange line = Hourly precipitation from ERA5 (mm). Blue highlight = Duration of precipitation event. Green vertical line = Snow sampling time.

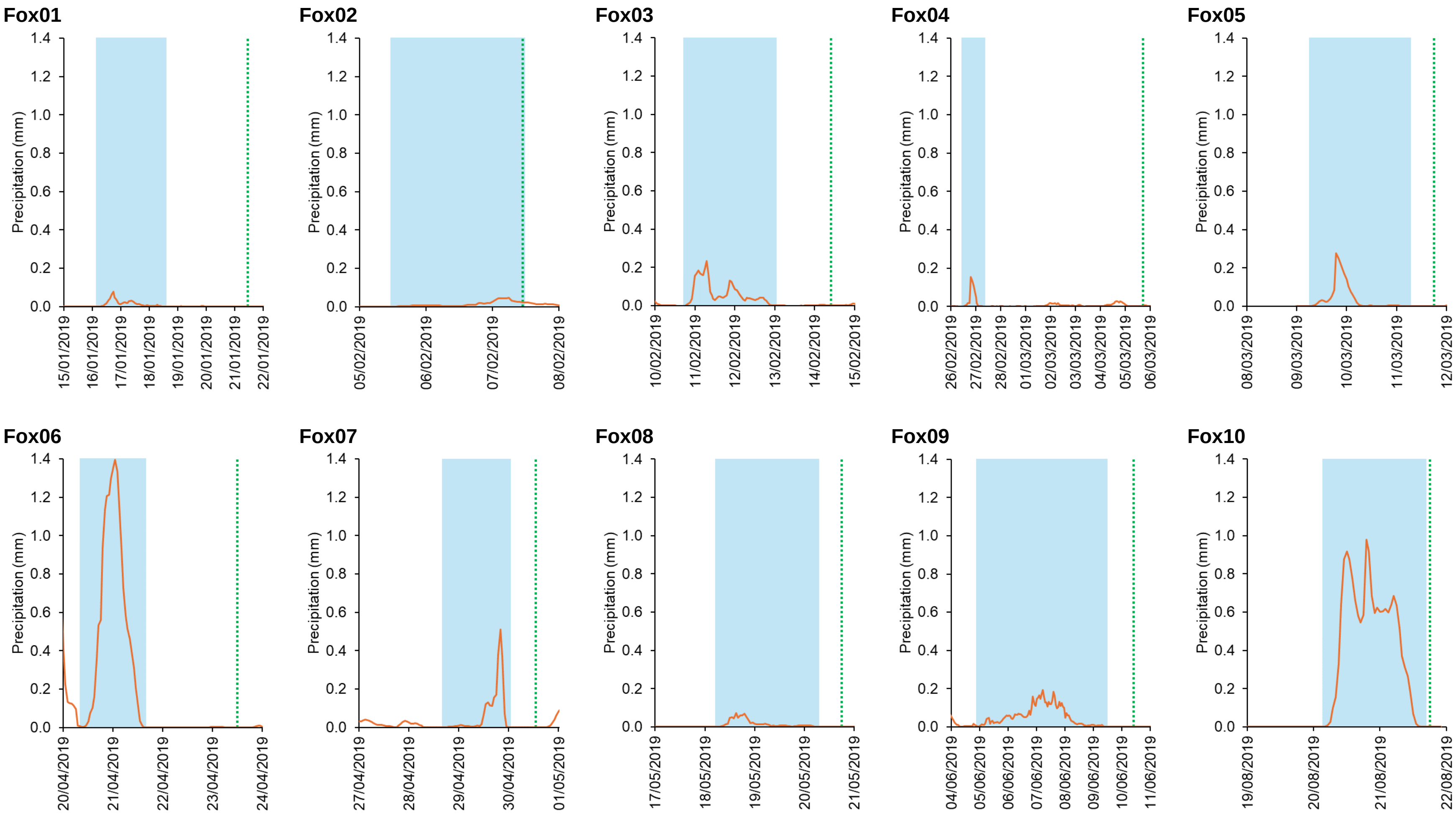

Figure S2c: Meteorological data for Foxfonna. Orange line = Hourly precipitation from ERA5 (mm). Blue highlight = duration of precipitation event. Green vertical line = Snow sampling time.

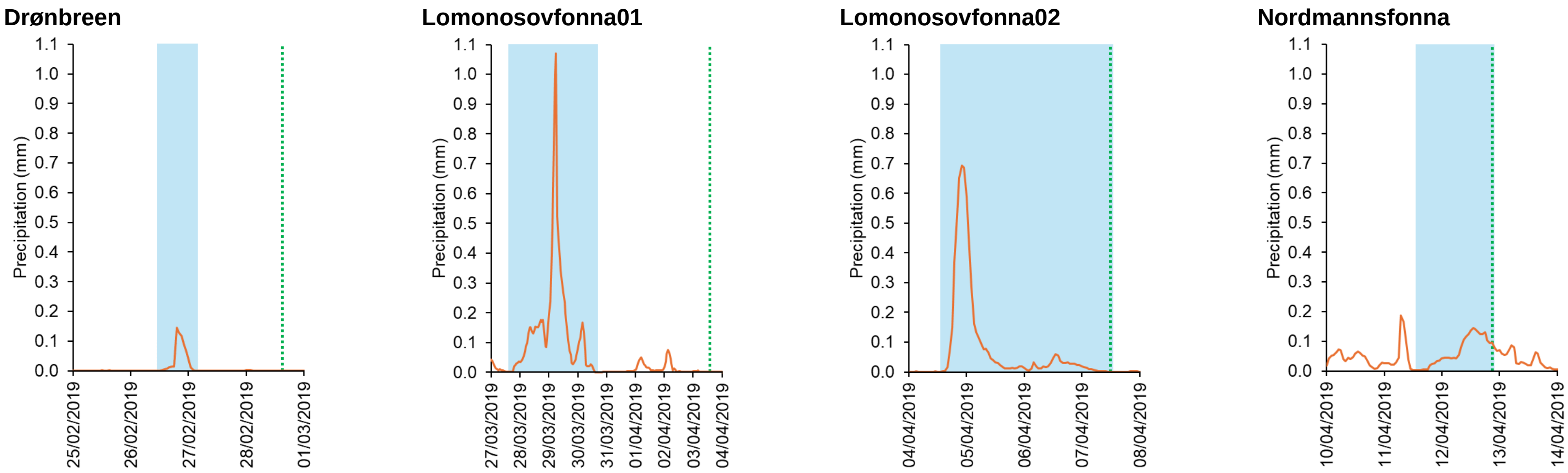

Figure S2d: Meteorological data at the Drønbreen, Lomonosovfonna and Nordmannsfonna reference sites. Orange line = Hourly precipitation from ERA5 (mm). Blue highlight = Duration of precipitation event. Green vertical line = Snow sampling time.

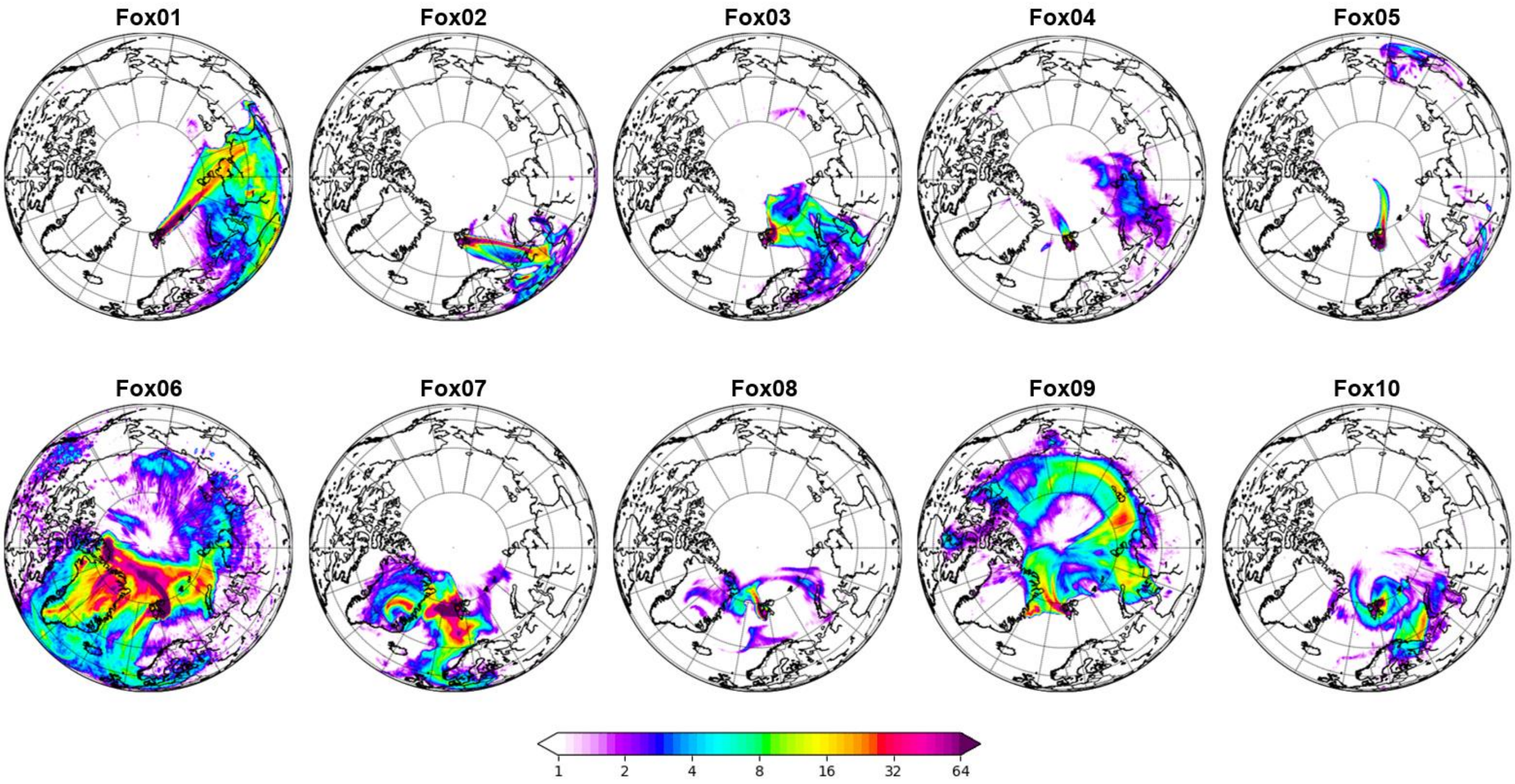

Figure S3a. FLEXPART backward airmass trajectory frequency plots (mm)

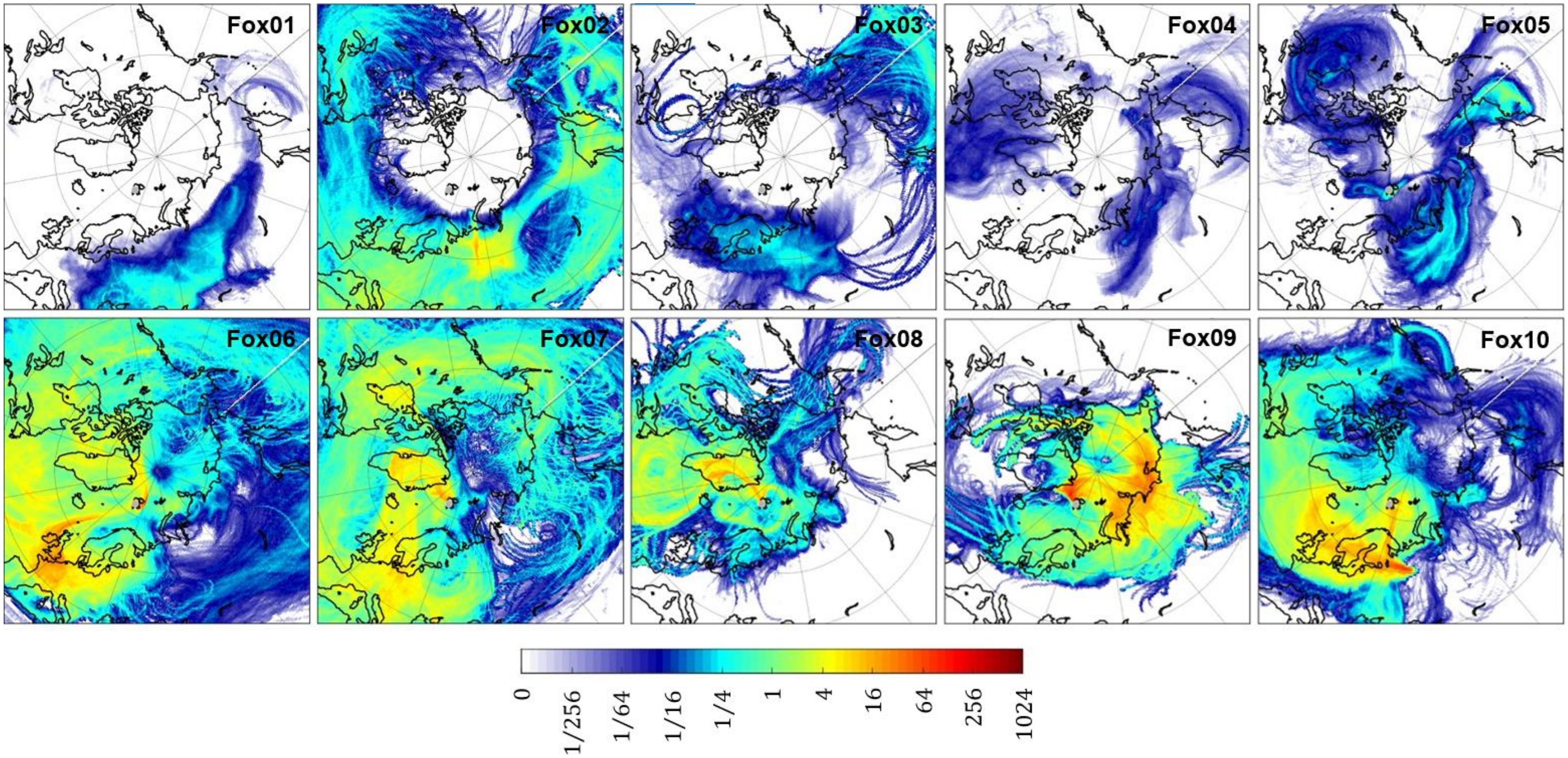

Figure S3b. FLEXPART backward airmass trajectory frequency plots weighted with downward UV radiation (TJ m^{-2})
